# Supplementary material for: Tilorone and Cridanimod Protect Mice and Show Antiviral Activity in Rats despite Absence of the Interferon-Inducing Effect in Rats
Source: Pharmaceuticals (Basel). 2022 May 17;15(5):617. doi: 10.3390/ph15050617 (PMC9143969; doi:10.3390/ph15050617)
Supplement: Supplementary file 1 [file pharmaceuticals-15-00617-s001.zip › pharmaceuticals-1691661-supplementary.pdf]

Supplementary Material

to

Tilorone and Cridanimod Protect Mice and Show Antiviral Activity in Rats Despite Absence of the Interferon-Inducing Effect in Rats

Viktoriya Keyer, Laura Syzdykova, Gulzat Zauatbayeva, Aigerim Zhulikeyeva, Yerlan Ramanculov, Alexandr V. Shustov*, Zarina Shulgau

National Center for Biotechnology, Korgalzhin hwy 13/5, Nur-Sultan 010000, Kazakhstan

***Correspondence**:
Alexandr V. Shustov, shustov@biocenter.kz, phone: +87029484554

**V.K.:** keer@biocenter.kz	ORCID 0000-0001-8885-2387
**L.S.:** syzdykova@biocenter.kz	ORCID 0000-0002-8889-1615
**G.Z.:** zauatbaeva@biocenter.kz	ORCID 0000-0003-1514-9302
**A.Zh.:** zhulikeeva@biocenter.kz	ORCID 0000-0001-6136-8093
**Y.R.:** ramanculov@biocenter.kz	ORCID 0000-0001-6786-3028
**A.V.S.**: shustov@biocenter.kz	ORCID 0000-0001-9880-9382
**Z.Sh.:** shulgau@biocenter.kz	ORCID 0000-0001-8148-0816

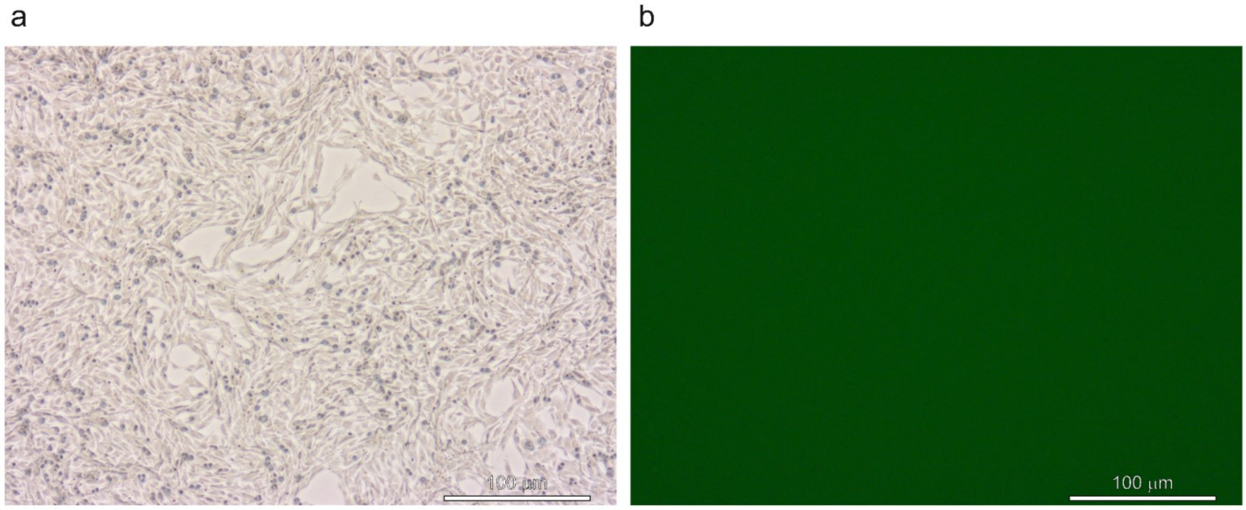

Figure S1. Control uninfected BHK-21 cells. **(a)** Image in white light. **(b)** Image in blue light to excite GFP. The same field as in panel **(a).** Magnification 50X.

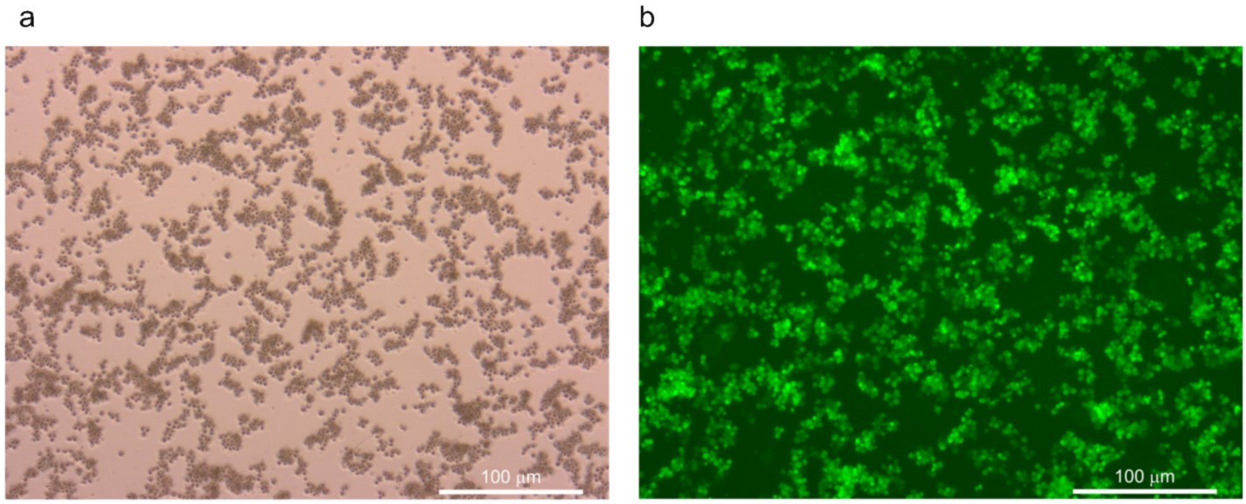

Figure S2. Pictures of the BHK-21 cell culture at 72 hours after transfection with a molecular infectious clone to rescue the cTC-83/TrD-GFP virus. **(a)** Image in white light. Cytopathic effect (CPE) is clearly visible. **(b)** GFP fluorescence in the same culture shown in panel **(a)**, the same microscopic field is shown in blue light. Magnification 50X.

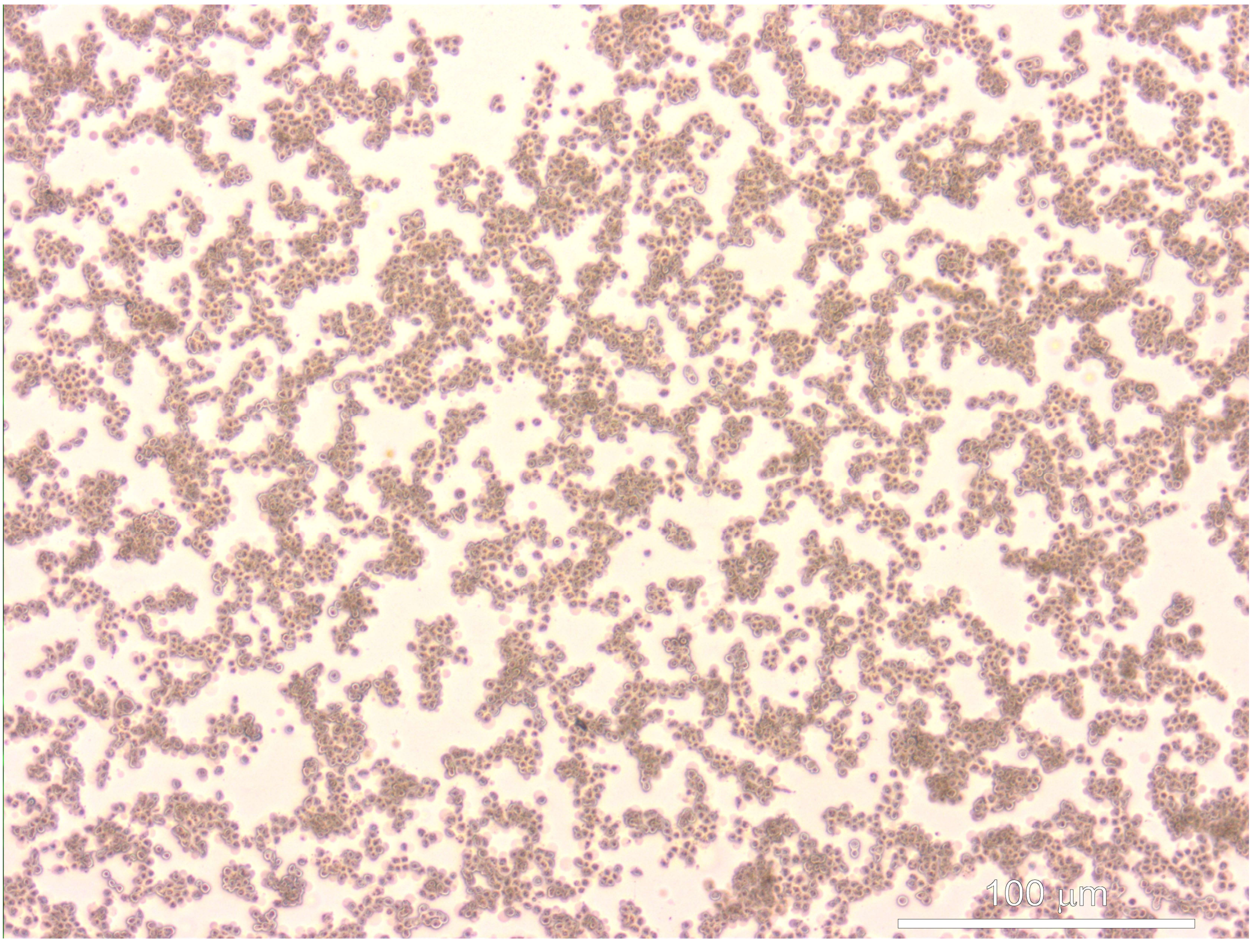

Figure S3. Cytopathic effect (CPE) in BHK-21 cells infected with the cTC-83 virus. Image in white light was taken on day 3 after transfection with a molecular infectious clone to rescue the cTC-83 virus. Magnification 50X.

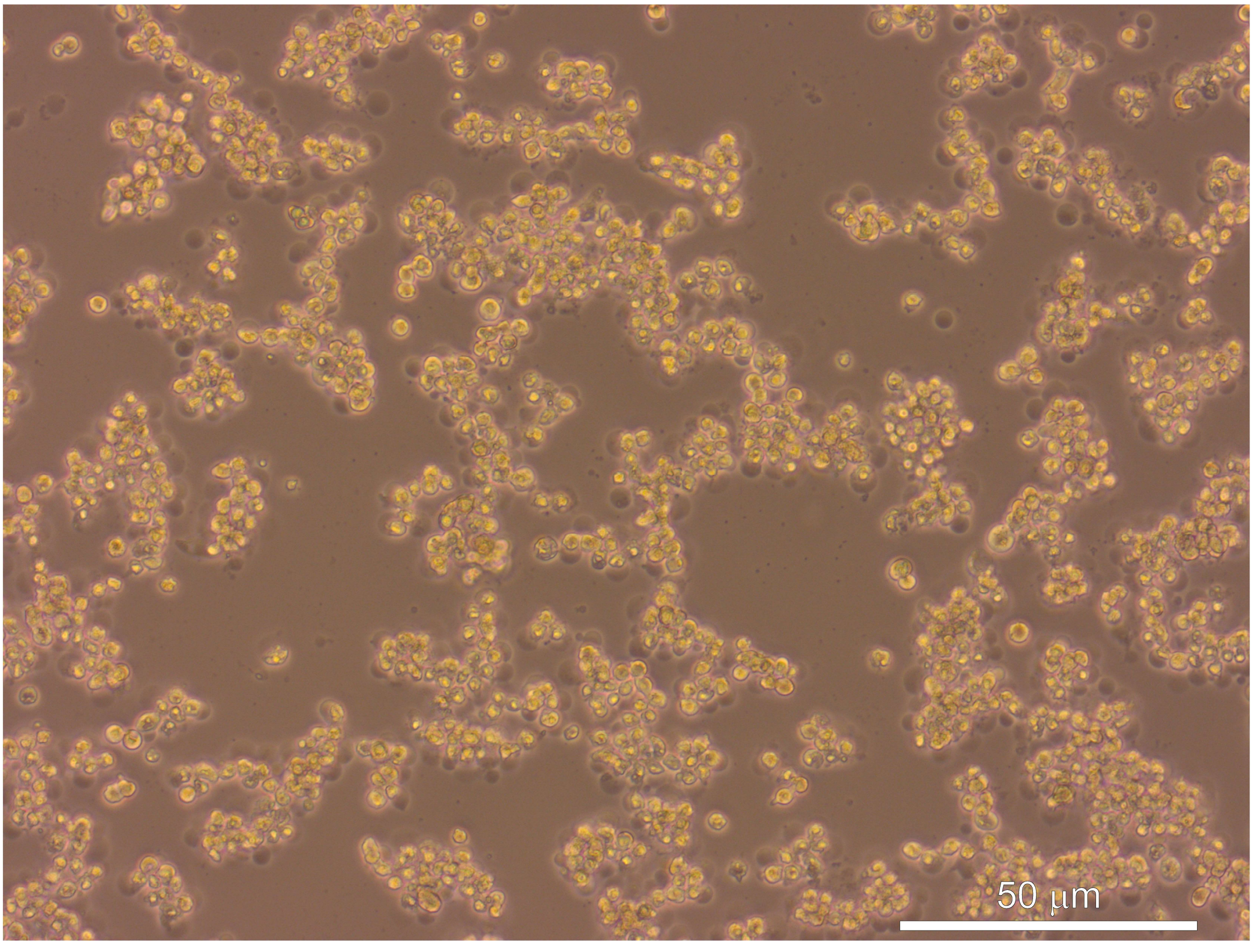

Figure S4. Cytopathic effect (CPE) in BHK-21 cells infected with the cTC-83/TrD virus. Image in white light was taken on day 3 after transfection with a molecular infectious clone to rescue the cTC-83/TrD virus. Magnification 100X.

Figure S5. Nucleotide sequence of the cTC-83 molecular infectious clone

```
LOCUS       cTC-83       16682 bp    DNA     circular     10-JAN-2022
SOURCE
  ORGANISM
FEATURES             Location/Qualifiers
     rep_origin      14366..14911
                     /vntifkey="33"
                     /label=p15A\origin\of\replication
     CDS             complement(15076..15936)
                     /vntifkey="4"
                     /label=Ap
     polyA_signal    11965..12550
                     /vntifkey="25"
                     /label=PolyA\signal\from\HGH\(human\growth\hormone)
     misc_feature    12635..12937
                     /vntifkey="21"
                     /label=SV40\ori
     promoter        16111..16682
                     /vntifkey="29"
                     /label=CMV\promoter
     misc_RNA        11473..11556
                     /vntifkey="53"
                     /label=Antigenomic\RBZ\HDV
     misc_feature    11965..4
                     /vntifkey="21"
                     /label=plasmid\vector
     CDS             8564..9832
                     /vntifkey="4"
                     /label=E2
     CDS             9833..10000
                     /product="6K protein"
                     /vntifkey="4"
                     /label=6k
                     /note="putative"
     CDS             8387..8563
                     /product="E3"
                     /function="incorporated into the envelope of some alpha
                     viruses"
                     /vntifkey="4"
                     /label=E3
                     /note="putative"
     CDS             5703..7523
                     /product="nsP4"
                     /function="replication"
                     /vntifkey="4"
                     /label=nsP4
                     /note="putative"
     CDS             4032..5702
                     /product="nsP3"
                     /function="replication"
                     /vntifkey="4"
```

/label=nsP3
/note="putative"
CDS 1650..4031
/product="nsP2"
/function="replication"
/vntifkey="4"
/label=nsP2
/note="putative"
CDS 45..1649
/product="nsP1"
/vntifkey="4"
/label=nsP1
/note="putative"
5'UTR 1..44
/vntifkey="52"
/label=5'UTR
promoter 7501..7545
/vntifkey="29"
/label=Subgenomic\promoter\(SP)
CDS 7562..8386
/vntifkey="4"
/label=C
CDS 10001..11326
/vntifkey="4"
/label=E1
CDS 13015..13806
/vntifkey="4"
/label=Neo
BASE COUNT 4481 a 4163 c 4211 g 3827 t
ORIGIN
1 ataggcggcg catgagagaa gcccagacca attacctacc caaaatggag aaagttcacg
61 ttgacatcga ggaagacagc ccattcctca gagctttgca gcggagcttc ccgcagtttg
121 aggtagaagc caagcaggtc actgataatg accatgctaa tgccagagcg ttttcgcatc
181 tggcttcaaa actgatcgaa acggaggtgg acccatccga cacgatcctt gacattggaa
241 gtgcgcccgc ccgcagaatg tattctaagc acaagtatca ttgtatctgt ccgatgagat
301 gtgcggaaga tccggacaga ttgtataagt atgcaactaa gctgaagaaa aactgtaagg
361 aaataactga taaggaattg gacaagaaaa tgaaggagct cgccgccgtc atgagcgacc
421 ctgacctgga aactgagact atgtgcctcc acgacgacga gtcgtgtcgc tacgaagggc
481 aagtcgctgt ttaccaggat gtatacgcgg ttgacggacc gacaagtctc tatcaccaag
541 ccaataaggg agttagagtc gcctactgga taggctttga caccacccct tttatgttta
601 agaacttggc tggagcatat ccatcatact ctaccaactg ggccgacgaa accgtgttaa

661 cggctcgtaa cataggccta tgcagctctg acgttatgga gcggtcacgt agagggatgt
721 ccattcttag aaagaagtat ttgaaaccat ccaacaatgt tctattctct gttggctcga
781 ccatctacca cgagaagagg gacttactga ggagctggca cctgccgtct gtatttcact
841 tacgtggcaa gcaaaattac acatgtcggt gtgagactat agttagttgc gacgggtacg
901 tcgttaaaag aatagctatc agtccaggcc tgtatgggaa gccttcaggc tatgctgcta
961 cgatgcaccg cgagggattc ttgtgctgca aagtgacaga cacattgaac ggggagaggg
1021 tctcttttcc cgtgtgcacg tatgtgccag ctacattgtg tgaccaaatg actggcatac
1081 tggcaacaga tgtcagtgcg gacgacgcgc aaaaactgct ggttgggctc aaccagcgta
1141 tagtcgtcaa cggtcgcacc cagagaaaca ccaataccat gaaaaattac cttttgcccg
1201 tagtggccca ggcatttgct aggtgggcaa aggaatataa ggaagatcaa gaagatgaaa
1261 ggccactagg actacgagat agacagttag tcatggggtg ttgttgggct tttagaaggc
1321 acaagataac atctatttat aagcgcccgg atacccaaac catcatcaaa gtgaacagcg
1381 atttccactc attcgtgctg cccaggatag gcagtaacac attggagatc gggctgagaa
1441 caagaatcag gaaaatgtta gaggagcaca aggagccgtc acctctcatt accgccgagg
1501 acgtacaaga agctaagtgc gcagccgatg aggctaagga ggtgcgtgaa gccgaggagt
1561 tgcgcgcagc tctaccacct ttggcagctg atgttgagga gcccactctg gaagccgatg
1621 tcgacttgat gttacaagag gctggggccg gctcagtgga gacacctcgt ggcttgataa
1681 aggttaccag ctacgatggc gaggacaaga tcggctctta cgctgtgctt tctccgcagg
1741 ctgtactcaa gagtgaaaaa ttatcttgca tccaccctct cgctgaacaa gtcatagtga
1801 taacacactc tggccgaaaa gggcgttatg ccgtggaacc ataccatggt aaagtagtgg
1861 tgccagaggg acatgcaata cccgtccagg actttcaagc tctgagtgaa agtgccacca
1921 ttgtgtacaa cgaacgtgag ttcgtaaaca ggtacctgca ccatattgcc acacatggag
1981 gagcgctgaa cactgatgaa gaatattaca aaactgtcaa gcccagcgag cacgacggcg
2041 aatacctgta cgacatcgac aggaaacagt gcgtcaagaa agaactagtc actgggctag
2101 ggctcacagg cgagctggtg gatcctccct tccatgaatt cgcctacgag agtctgagaa
2161 cacgaccagc cgctccttac caagtaccaa ccataggggt gtatggcgtg ccaggatcag

2221 gcaagtctgg catcattaaa agcgcagtca ccaaaaaaga tctagtggtg agcgccaaga
2281 aagaaaactg tgcagaaatt ataagggacg tcaagaaaat gaaagggctg gacgtcaatg
2341 ccagaactgt ggactcagtg ctcttgaatg gatgcaaaca ccccgtagag accctgtata
2401 ttgacgaagc ttttgcttgt catgcaggta ctctcagagc gctcatagcc attataagac
2461 ctaaaaaggc agtgctctgc ggggatccca aacagtgcgg tttttttaac atgatgtgcc
2521 tgaaagtgca ttttaaccac gagatttgca cacaagtctt ccacaaaagc atctctcgcc
2581 gttgcactaa atctgtgact tcggtcgtct caaccttgtt ttacgacaaa aaaatgagaa
2641 cgacgaatcc gaaagagact aagattgtga ttgacactac cggcagtacc aaacctaagc
2701 aggacgatct cattctcact tgtttcagag ggtgggtgaa gcagttgcaa atagattaca
2761 aaggcaacga aataatgacg gcagctgcct ctcaagggct gacccgtaaa ggtgtgtatg
2821 ccgttcggta caaggtgaat gaaaatcctc tgtacgcacc cacctcagaa catgtgaacg
2881 tcctactgac ccgcacggag gaccgcatcg tgtggaaaac actagccggc gacccatgga
2941 taaaaacact gactgccaag taccctggga atttcactgc cacgatagag gagtggcaag
3001 cagagcatga tgccatcatg aggcacatct tggagagacc ggaccctacc gacgtcttcc
3061 agaataaggc aaacgtgtgt tgggccaagg ctttagtgcc ggtgctgaag accgctggca
3121 tagacatgac cactgaacaa tggaacactg tggattattt tgaaacggac aaagctcact
3181 cagcagagat agtattgaac caactatgcg tgaggttctt tggactcgat ctggactccg
3241 gtctattttc tgcacccact gttccgttat ccattaggaa taatcactgg gataactccc
3301 cgtcgcctaa catgtacggg ctgaataaag aagtggtccg tcagctctct cgcaggtacc
3361 cacaactgcc tcgggcagtt gccactggaa gagtctatga catgaacact ggtacactgc
3421 gcaattatga tccgcgcata aacctagtac ctgtaaacag aagactgcct catgctttag
3481 tcctccacca taatgaacac ccacagagtg acttttcttc attcgtcagc aaattgaagg
3541 gcagaactgt cctggtggtc ggggaaaagt tgtccgtccc aggcaaaatg gttgactggt
3601 tgtcagaccg gcctgaggct accttcagag ctcggctgga tttaggcatc ccaggtgatg
3661 tgcccaaata tgacataata tttgttaatg tgaggacccc atataaatac catcactatc
3721 agcagtgtga agaccatgcc attaagctta gcatgttgac caagaaagct tgtctgcatc

3781 tgaatcccgg cggaacctgt gtcagcatag gttatggtta cgctgacagg gccagcgaaa
3841 gcatcattgg tgctatagcg cggcagttca agttttcccg ggtatgcaaa ccgaaatcct
3901 cacttgaaga gacggaagtt ctgtttgtat tcattgggta cgatcgcaag gcccgtacgc
3961 acaatcctta caagctttca tcaaccttga ccaacattta tacaggttcc agactccacg
4021 aagccggatg tgcaccctca tatcatgtgg tgcgagggga tattgccacg gccaccgaag
4081 gagtgattat aaatgctgct aacagcaaag gacaacctgg cggagggggtg tgcggagcgc
4141 tgtataagaa attcccggaa agcttcgatt tacagccgat cgaagtagga aaagcgcgac
4201 tggtcaaagg tgcagctaaa catatcattc atgccgtagg accaaacttc aacaaagttt
4261 cggaggttga aggtgacaaa cagttggcag aggcttatga gtccatcgct aagattgtca
4321 acgataacaa ttacaagtca gtagcgattc cactgttgtc caccggcatc ttttccggga
4381 acaaagatcg actaacccaa tcattgaacc atttgctgac agctttagac accactgatg
4441 cagatgtagc catatactgc agggacaaga aatgggaaat gactctcaag gaagcagtgg
4501 ctaggagaga agcagtggag gagatatgca tatccgacga ctcttcagtg acagaacctg
4561 atgcagagct ggtgagggtg catccgaaga gttctttggc tggaaggaag ggctacagca
4621 caagcgatgg caaaactttc tcatatttgg aagggaccaa gtttcaccag gcggccaagg
4681 atatagcaga aattaatgcc atgtggcccg ttgcaacgga ggccaatgag caggtatgca
4741 tgtatatcct cggagaaagc atgagcagta ttaggtcgaa atgccccgtc gaagagtcgg
4801 aagcctccac accacctagc acgctgcctt gcttgtgcat ccatgccatg actccagaaa
4861 gagtacagcg cctaaaagcc tcacgtccag aacaaattac tgtgtgctca tcctttccat
4921 tgccgaagta tagaatcact ggtgtgcaga agatccaatg ctcccagcct atattgttct
4981 caccgaaagt gcctgcgtat attcatccaa ggaagtatct cgtggaaaca ccaccggtag
5041 acgagactcc ggagccatcg gcagagaacc aatccacaga ggggacacct gaacaaccac
5101 cacttataac cgaggatgag accaggacta gaacgcctga gccgatcatc atcgaagagg
5161 aagaagagga tagcataagt ttgctgtcag atggcccgac ccaccaggtg ctgcaagtcg
5221 aggcagacat tcacgggccg ccctctgtat ctagctcatc ctggtccatt cctcatgcat
5281 ccgactttga tgtggacagt ttatccatac ttgacaccct ggagggagct agcgtgacca

5341 gcggggcaac gtcagccgag actaactctt acttcgcaaa gagtatggag tttctggcgc
5401 gaccggtgcc tgcgcctcga acagtattca ggaaccctcc acatcccgct ccgcgcacaa
5461 gaacaccgtc acttgcaccc agcagggcct gctcgagaac cagcctagtt tccaccccgc
5521 caggcgtgaa tagggtgatc actagagagg agctcgaggc gcttaccccg tcacgcactc
5581 ctagcaggtc ggtctcgaga accagcctgg tctccaaccc gccaggcgta aatagggtga
5641 ttacaagaga ggagtttgag gcgttcgtag cacaacaaca atgacggttt gatgcgggtg
5701 catacatctt ttcctccgac accggtcaag ggcatttaca acaaaaatca gtaaggcaaa
5761 cggtgctatc cgaagtggtg ttggagagga ccgaattgga gatttcgtat gccccgcgcc
5821 tcgaccaaga aaaagaagaa ttactacgca agaaattaca gttaaatccc acacctgcta
5881 acagaagcag ataccagtcc aggaaggtgg agaacatgaa agccataaca gctagacgta
5941 ttctgcaagg cctagggcat tatttgaagg cagaaggaaa agtggagtgc taccgaaccc
6001 tgcatcctgt tcctttgtat tcatctagtg tgaaccgtgc cttttcaagc cccaaggtcg
6061 cagtggaagc ctgtaacgcc atgttgaaag agaactttcc gactgtggct tcttactgta
6121 ttattccaga gtacgatgcc tatttggaca tggttgacgg agcttcatgc tgcttagaca
6181 ctgccagttt ttgccctgca aagctgcgca gctttccaaa gaaacactcc tatttggaac
6241 ccacaatacg atcggcagtg ccttcagcga tccagaacac gctccagaac gtcctggcag
6301 ctgccacaaa aagaaattgc aatgtcacgc aaatgagaga attgcccgta ttggattcgg
6361 cggcctttaa tgtggaatgc ttcaagaaat atgcgtgtaa taatgaatat tgggaaacgt
6421 ttaaagaaaa ccccatcagg cttactgaag aaaacgtggt aaattacatt accaaattaa
6481 aaggaccaaa agctgctgct ctttttgcga agacacataa tttgaatatg ttgcaggaca
6541 taccaatgga caggtttgta atggacttaa agagagacgt gaaagtgact ccaggaacaa
6601 aacatactga agaacggccc aaggtacagg tgatccaggc tgccgatccg ctagcaacag
6661 cgtatctgtg cggaatccac cgagagctgg ttaggagatt aaatgcggtc ctgcttccga
6721 acattcatac actgtttgat atgtcggctg aagactttga cgctattata gccgagcact
6781 tccagcctgg ggattgtgtt ctggaaactg acatcgcgtc gtttgataaa agtgaggacg
6841 acgccatggc tctgaccgcg ttaatgattc tggaagactt aggtgtggac gcagagctgt

6901 tgacgctgat tgaggcggct ttcggcgaaa tttcatcaat acatttgccc actaaaacta
6961 aatttaaatt cggagccatg atgaaatctg gaatgttcct cacactgttt gtgaacacag
7021 tcattaacat tgtaatcgca agcagagtgt tgagagaacg gctaaccgga tcaccatgtg
7081 cagcattcat tggagatgac aatatcgtga aaggagtcaa atcggacaaa ttaatggcag
7141 acaggtgcgc cacctggttg aatatggaag tcaagattat agatgctgtg gtgggcgaga
7201 aagcgcctta tttctgtgga gggtttattt tgtgtgactc cgtgaccggc acagcgtgcc
7261 gtgtggcaga ccccctaaaa aggctgttta agcttggcaa acctctggca gcagacgatg
7321 aacatgatga tgacaggaga agggcattgc atgaagagtc aacacgctgg aaccgagtgg
7381 gtattctttc agagctgtgc aaggcagtag aatcaaggta tgaaaccgta ggaacttcca
7441 tcatagttat ggccatgact actctagcta gcagtgttaa atcattcagc tacctgagag
7501 gggcccctat aactctctac ggctaacctg aatggactac gacatagtct agtccgccaa
7561 gatgttcccg ttccagccaa tgtatccgat gcagccaatg ccctatcgca acccgttcgc
7621 ggccccgcgc aggccctggt tccccagaac cgaccctttt ctggcgatgc aggtgcagga
7681 attaacccgc tcgatggcta acctgacgtt caagcaacgc cgggacgcgc cacctgaggg
7741 gccatccgct aagaaaccga agaaggaggc ctcgcaaaaa cagaaagggg gaggccaagg
7801 gaagaagaag aagaaccaag ggaagaagaa ggctaagaca gggccgccta atccgaaggc
7861 acagaatgga aacaagaaga agaccaacaa gaaaccaggc aagagacagc gcatggtcat
7921 gaaattggaa tctgacaaga cgttcccaat catgttggaa gggaagataa acggctacgc
7981 ttgtgtggtc ggagggaagt tattcaggcc gatgcatgtg gaaggcaaga tcgacaacga
8041 cgttctggcc gcgcttaaga cgaagaaagc atccaaatac gatcttgagt atgcagatgt
8101 gccacagaac atgcgggccg atacattcaa atacacccat gagaaacccc aaggctatta
8161 cagctggcat catggagcag tccaatatga aaatgggcgt ttcacggtgc cgaaaggagt
8221 tggggccaag ggagacagcg gacgacccat tctggataac cagggacggg tggtcgctat
8281 tgtgctggga ggtgtgaatg aaggatctag gacagccctt tcagtcgtca tgtggaacga
8341 gaagggagtt accgtgaagt atactccgga gaactgcgag caatggtcac tagtgaccac
8401 catgtgtctg ctcgccaatg tgacgttccc atgtgctcaa ccaccaattt gctacgacag

8461 aaaaccagca gagactttgg ccatgctcag cgttaacgtt gacaacccgg gctacgatga
8521 gctgctggaa gcagctgtta agtgccccgg aaggaaaagg agatccaccg aggagctgtt
8581 taatgagtat aagctaacgc gcccttacat ggccagatgc atcagatgtg cagttgggag
8641 ctgccatagt ccaatagcaa tcgaggcagt aaagagcgac gggcacgacg gttatgttag
8701 acttcagact tcctcgcagt atggcctgga ttcctccggc aacttaaagg gcaggaccat
8761 gcggtatgac atgcacggga ccattaaaga gataccacta catcaagtgt cactctatac
8821 atctcgcccg tgtcacattg tggatgggca cggttatttc ctgcttgcca ggtgcccggc
8881 aggggactcc atcaccatgg aatttaagaa agattccgtc agacactcct gctcggtgcc
8941 gtatgaagtg aaatttaatc ctgtaggcag agaactctat actcatcccc cagaacacgg
9001 agtagagcaa gcgtgccaag tctacgcaca tgatgcacag aacagaggag cttatgtcga
9061 gatgcacctc ccgggctcag aagtggacag cagtttggtt tccttgagcg gcagttcagt
9121 caccgtgaca cctcctgatg ggactagcgc cctggtggaa tgcgagtgtg gcggcacaaa
9181 gatctccgag accatcaaca agacaaaaca gttcagccag tgcacaaaga aggagcagtg
9241 cagagcatat cggctgcaga acgataagtg ggtgtataat tctgacaaac tgcccaaagc
9301 agcgggagcc accttaaaag gaaaactgca tgtcccattc ttgctggcag acggcaaatg
9361 caccgtgcct ctagcaccag aacctatgat aaccttcggt ttcagatcag tgtcactgaa
9421 actgcaccct aagaatccca catatctaat cacccgccaa cttgctgatg agcctcacta
9481 cacgcacgag ctcatatctg aaccagctgt taggaatttt accgtcaccg aaaaagggtg
9541 ggagtttgta tggggaaacc acccgccgaa aaggttttgg gcacaggaaa cagcacccgg
9601 aaatccacat gggctaccgc acgaggtgat aactcattat taccacagat accctatgtc
9661 caccatcctg ggtttgtcaa tttgtgccgc cattgcaacc gtttccgttg cagcgtctac
9721 ctggctgttt tgcagatcta gagttgcgtg cctaactcct taccggctaa cacctaacgc
9781 taggatacca ttttgtctgg ctgtgctttg ctgcgcccgc actgcccggg ccgagaccac
9841 ctgggagtcc ttggatcacc tatggaacaa taaccaacag atgttctgga ttcaattgct
9901 gatccctctg gccgccttga tcgtagtgac tcgcctgctc aggtgcgtgt gctgtgtcgt
9961 gcctttttta gtcatggccg gcgccgcagg cgccggcgcc tacgagcacg cgaccacgat

10021 gccgagccaa gcgggaatct cgtataacac tatagtcaac agagcaggct acgcaccact
10081 ccctatcagc ataacaccaa caaagatcaa gctgatacct acagtgaact tggagtacgt
10141 cacctgccac tacaaaacag gaatggattc accagccatc aaatgctgcg gatctcagga
10201 atgcactcca acttacaggc ctgatgaaca gtgcaaagtc ttcacagggg tttacccgtt
10261 catgtggggt ggtgcatatt gcttttgcga cactgagaac acccaagtca gcaaggccta
10321 cgtaatgaaa tctgacgact gccttgcgga tcatgctgaa gcatataaag cgcacacagc
10381 ctcagtgcag gcgttcctca acatcacagt gggagaacac tctattgtga ctaccgtgta
10441 tgtgaatgga gaaactcctg tgaatttcaa tggggtcaaa ataactgcag gtccgctttc
10501 cacagcttgg acaccctttg atcgcaaaat cgtgcagtat gccggggaga tctataatta
10561 tgattttcct gagtatgggg caggacaacc aggagcattt ggagatatac aatccagaac
10621 agtctcaagc tctgatctgt atgccaatac caacctagtg ctgcagagac ccaaagcagg
10681 agcgatccac gtgccataca ctcaggcacc ttcgggtttt gagcaatgga agaaagataa
10741 agctccatca ttgaaattta ccgccccttt cggatgcgaa atatatacaa accccattcg
10801 cgccgaaaac tgtgctgtag ggtcaattcc attagccttt gacattcccg acgccttgtt
10861 caccagggtg tcagaaacac cgacactttc agcggccgaa tgcactctta acgagtgcgt
10921 gtattcttcc gactttggtg ggatcgccac ggtcaagtac tcggccagca agtcaggcaa
10981 gtgcgcagtc catgtgccat cagggactgc taccctaaaa gaagcagcag tcgagctaac
11041 cgagcaaggg tcggcgacta tccatttctc gaccgcaaat atccacccgg agttcaggct
11101 ccaaatatgc acatcatatg ttacgtgcaa aggtgattgt caccccccga aagaccatat
11161 tgtgacacac cctcagtatc acgcccaaac atttacagcc gcggtgtcaa aaaccgcgtg
11221 gacgtggtta acatccctgc tgggaggatc agccgtaatt attataattg gcttggtgct
11281 ggctactatt gtggccatgt acgtgctgac caaccagaaa cataattgaa tacagcagca
11341 attggcaagc tgcttacata gaactcgcgg cgattggcat gccgccttaa aatttttatt
11401 ttattttct tttcttttcc gaatcggatt ttgtttttaa tatttcaaaa aaaaaaaaaa
11461 aaaaaaaaaa aagggtcggc atggcatctc cacctcctcg cggtccgacc tgggcatccg
11521 aaggaggacg cacgtccact cggatggcta agggagagcc acgagctcct cgacagatca

11581 taatcagcca taccacattt gtagaggttt tacttgcttt aaaaaacctc ccacacctcc
11641 ccctgaacct gaaacataaa atgaatgcaa ttgttgttgt taacttgttt attgcagctt
11701 ataatggtta caaataaagc aatagcatca caaatttcac aaataaagca tttttttcac
11761 tgcattctag ttgtggtttg tccaaactca tcaagatacg cgtacgcggc cccatgttcg
11821 ccttccgccg cgtggaggag gatcacagca acaccgagct gggcatcgtg gagtaccagc
11881 acgccttcaa gaccccggat gcagatgccg gtgaagaaag agtttaaacg gccggccgcg
11941 gtcatagctg tttcctgaac agatcccggg tggcatccct gtgacccctc cccagtgcct
12001 ctcctggccc tggaagttgc cactccagtg cccaccagcc ttgtcctaat aaaattaagt
12061 tgcatcattt tgtctgacta ggtgtccttc tataatatta tggggtggag gggggtggta
12121 tggagcaagg ggcaagttgg gaagacaacc tgtagggcct gcggggtcta ttgggaacca
12181 agctggagtg cagtggcaca atcttggctc actgcaatct ccgcctcctg ggttcaagcg
12241 attctcctgc ctcagcctcc cgagttgttg ggattccagg catgcatgac caggctcagc
12301 taatttttgt ttttttggta gagacggggt ttcaccatat tggccaggct ggtctccaac
12361 tcctaatctc aggtgatcta cccaccttgg cctcccaaat tgctgggatt acaggcgtga
12421 accactgctc ccttccctgt ccttctgatt ttaaaataac tataccagca ggaggacgtc
12481 cagacacagc ataggctacc tggccatgcc caaccggtgg gacatttgag ttgcttgctt
12541 ggcactgtcc tctcatgcgt tgggtccact cagtagatgc ctgttgaatt gggtacgcgg
12601 ccagcttggc tgtggaatgt gtgtcagtta gggtgtggaa agtccccagg ctccccagca
12661 ggcagaagta tgcaaagcat gcatctcaat tagtcagcaa ccaggtgtgg aaagtcccca
12721 ggctccccag caggcagaag tatgcaaagc atgcatctca attagtcagc aaccatagtc
12781 ccgcccctaa ctccgcccat cccgccccta actccgccca gttccgccca ttctccgccc
12841 catggctgac taattttttt tatttatgca gaggccgagg ccgcctcggc ctctgagcta
12901 ttccagaagt agtgaggagg cttttttgga ggcctaggct tttgcaaaaa gctcccggga
12961 gcttgtatat ccattttcgg atctgatcaa gagacaggat gaggatcgtt tcgcatgatt
13021 gaacaagatg gattgcacgc aggttctccg gccgcttggg tggagaggct attcggctat
13081 gactgggcac aacagacaat cggctgctct gatgccgccg tgttccggct gtcagcgcag

13141 gggcgcccgg ttctttttgt caagaccgac ctgtccggtg ccctgaatga actgcaggac
13201 gaggcagcgc ggctatcgtg gctggccacg acgggcgttc cttgcgcagc tgtgctcgac
13261 gttgtcactg aagcgggaag ggactggctg ctattgggcg aagtgccggg gcaggatctc
13321 ctgtcatctc accttgctcc tgccgagaaa gtatccatca tggctgatgc aatgcggcgg
13381 ctgcatacgc ttgatccggc tacctgccca ttcgaccacc aagcgaaaca tcgcatcgag
13441 cgagcacgta ctcggatgga agccggtctt gtcgatcagg atgatctgga cgaagagcat
13501 caggggctcg cgccagccga actgttcgcc aggctcaagg cgcgcatgcc cgacggcgag
13561 gatctcgtcg tgacccatgg cgatgcctgc ttgccgaata tcatggtgga aaatggccgc
13621 ttttctggat tcatcgactg tggccggctg ggtgtggccg accgctatca ggacatagcg
13681 ttggctaccc gtgatattgc tgaagagctt ggcggcgaat gggctgaccg cttcctcgtg
13741 ctttacggta tcgccgctcc cgattcgcag cgcatcgcct tctatcgcct tcttgacgag
13801 ttcttctgag cgggactctg gggttcgaaa tgaccgacca agcgacgccc aacctgccat
13861 cacgagattt cgattccacc gccgccttct atgaaaggtt gggcttcgga atcgttttcc
13921 gggacgccgg ctggatgatc ctccagcgcg gggatctcat gctggagttc ttcgcccacc
13981 ccaacttgtt tattgcagct tataatggtt acaaataaag caatagcatc acaaatttca
14041 caaataaagc atttttttca ctgcattcta gttgtggttt gtccaaactc atcaatgtat
14101 cttatcatgt ctgtatactg gcttactatg ttggcactga tgagggtgtc agtgaagtgc
14161 ttcatgtggc aggagaaaaa aggctgcacc ggtgcgtcag cagaatatgt gatacaggat
14221 atattccgct tcctcgctca ctgactcgct acgctcggtc gttcgactgc ggcgagcgga
14281 aatggcttac gaacggggcg gagatttcct ggaagatgcc aggaagatac ttaacaggga
14341 agtgagaggg ccgcggcaaa gccgtttttc cataggctcc gcccccctga caagcatcac
14401 gaaatctgac gctcaaatca gtggtggcga aacccgacag gactataaag ataccaggcg
14461 tttccccctgg cggctccctc gtgcgctctc ctgttcctgc ctttcggttt accggtgtca
14521 ttccgctgtt atggccgcgt ttgtctcatt ccacgcctga cactcagttc cgggtaggca
14581 gttcgctcca agctggactg tatgcacgaa ccccccgttc agtccgaccg ctgcgcctta
14641 tccggtaact atcgtcttga gtccaacccg gaaagacatg caaaagcacc actggcagca

14701 gccactggta attgatttag aggagttagt cttgaagtca tgcgccggtt aaggctaaac
14761 tgaaaggaca agttttggtg actgcgctcc tccaagccag ttacctcggt tcaaagagtt
14821 ggtagctcag agaaccttcg aaaaaccgcc ctgcaaggcg gttttttcgt tttcagagca
14881 agagattacg cgcagaccaa aacgatctca agaagatcat cttattaagg ggtctgacgc
14941 tcagtggaac gaaaactcac gttaagggat tttggtcatg agattatcaa aaaggatctt
15001 cacctagatc cttttaaatt aaaaatgaag ttttaaatca atctaaagta tatatgagta
15061 aacttggtct gacagttacc aatgcttaat cagtgaggca cctatctcag cgatctgtct
15121 atttcgttca tccatagttg cctgactccc cgtcgtgtag ataactacga tacgggaggg
15181 cttaccatct ggccccagtg ctgcaatgat accgcgagac ccacgctcac cggctccaga
15241 tttatcagca ataaaccagc cagccggaag ggccgagcgc agaagtggtc ctgcaacttt
15301 atccgcctcc atccagtcta ttaattgttg ccgggaagct agagtaagta gttcgccagt
15361 taatagtttg cgcaacgttg ttgccattgc tgcaggcatc gtggtgtcac gctcgtcgtt
15421 tggtatggct tcattcagct ccggttccca acgatcaagg cgagttacat gatcccccat
15481 gttgtgcaaa aaagcggtta gctccttcgg tcctccgatc gttgtcagaa gtaagttggc
15541 cgcagtgtta tcactcatgg ttatggcagc actgcataat tctcttactg tcatgccatc
15601 cgtaagatgc ttttctgtga ctggtgagta ctcaaccaag tcattctgag aatagtgtat
15661 gcggcgaccg agttgctctt gcccggcgtc aacacgggat aataccgcgc cacatagcag
15721 aactttaaaa gtgctcatca ttggaaaacg ttcttcgggg cgaaaactct caaggatctt
15781 accgctgttg agatccagtt cgatgtaacc cactcgtgca cccaactgat cttcagcatc
15841 ttttactttc accagcgttt ctgggtgagc aaaaacagga aggcaaaatg ccgcaaaaaa
15901 gggaataagg gcgacacgga aatgttgaat actcatactc ttcctttttc aatattattg
15961 aagcatttat cagggttatt gtctcatgag cggatacata tttgaatgta tttagaaaaa
16021 taaacaaata ggggttccgc gcacatttcc ccgaaaagtg ccacctgacg tgtcgacgcg
16081 gccgcacatt gattattgac tagttattaa tagtaatcaa ttacggggtc attagttcat
16141 agcccatata tggagttccg cgttacataa cttacggtaa atggcccgcc tggctgaccg
16201 cccaacgacc cccgcccatt gacgtcaata atgacgtatg ttcccatagt aacgccaata

16261 gggactttcc attgacgtca atgggtggag tatttacggt aaactgccca cttggcagta
16321 catcaagtgt atcatatgcc aagtccgccc cctattgacg tcaatgacgg taaatggccc
16381 gcctggcatt atgcccagta catgacctta cgggactttc ctacttggca gtacatctac
16441 gtattagtca tcgctattac catggtgatg cggttttggc agtacaccaa tgggcgtgga
16501 tagcggtttg actcacgggg atttccaagt ctccacccca ttgacgtcaa tgggagtttg
16561 ttttggcacc aaaatcaacg ggactttcca aaatgtcgta ataaccccgc cccgttgacg
16621 caaatgggcg gtaggcgtgt acggtgggag gtctatataa gcagagctcg tttagtgaac
16681 cg
//

Figure S6. Nucleotide sequence of the cTC-83/TrD molecular infectious clone

```
LOCUS       cTC-83_TrD       16682 bp    DNA     circular     10-JAN-2022
SOURCE
  ORGANISM
FEATURES             Location/Qualifiers
     rep_origin      14366..14911
                     /vntifkey="33"
                     /label=p15A\origin\of\replication
     CDS             complement(15076..15936)
                     /vntifkey="4"
                     /label=Ap
     polyA_signal    11965..12550
                     /vntifkey="25"
                     /label=PolyA\signal\from\HGH\(human\growth\hormone)
     misc_feature    12635..12937
                     /vntifkey="21"
                     /label=SV40\ori
     promoter        16111..16682
                     /vntifkey="29"
                     /label=CMV\promoter
     misc_RNA        11473..11556
                     /vntifkey="53"
                     /label=Antigenomic\RBZ\HDV
     misc_feature    11965..4
                     /vntifkey="21"
                     /label=plasmid\vector
     CDS             8564..9832
                     /vntifkey="4"
                     /label=E2
     CDS             9833..10000
                     /product="6K protein"
                     /vntifkey="4"
                     /label=6k
                     /note="putative"
     CDS             8387..8563
                     /product="E3"
                     /function="incorporated into the envelope of some alpha
                     viruses"
                     /vntifkey="4"
                     /label=E3
                     /note="putative"
     CDS             5703..7523
                     /product="nsP4"
                     /function="replication"
                     /vntifkey="4"
                     /label=nsP4
                     /note="putative"
     CDS             4032..5702
                     /product="nsP3"
                     /function="replication"
                     /vntifkey="4"
```

/label=nsP3
/note="putative"
CDS 1650..4031
/product="nsP2"
/function="replication"
/vntifkey="4"
/label=nsP2
/note="putative"
CDS 45..1649
/product="nsP1"
/vntifkey="4"
/label=nsP1
/note="putative"
5'UTR 1..44
/vntifkey="52"
/label=5'UTR
promoter 7501..7545
/vntifkey="29"
/label=Subgenomic\promoter\(SP)
CDS 7562..8386
/vntifkey="4"
/label=C
CDS 10001..11326
/vntifkey="4"
/label=E1
CDS 13015..13806
/vntifkey="4"
/label=Neo
mutation 3..3
/vntifkey="62"
/label=A->G\(TrD)
mutation 8922..8922
/vntifkey="62"
/label=G->C\(Arg->Tre)\(TrD)
BASE COUNT 4480 a 4164 c 4211 g 3827 t
ORIGIN
1 atgggcggcg catgagagaa gcccagacca attacctacc caaaatggag
aaagttcacg
61 ttgacatcga ggaagacagc ccattcctca gagctttgca gcggagcttc
ccgcagtttg
121 aggtagaagc caagcaggtc actgataatg accatgctaa tgccagagcg
ttttcgcatc
181 tggcttcaaa actgatcgaa acggaggtgg acccatccga cacgatcctt
gacattggaa
241 gtgcgcccgc ccgcagaatg tattctaagc acaagtatca ttgtatctgt
ccgatgagat
301 gtgcggaaga tccggacaga ttgtataagt atgcaactaa gctgaagaaa
aactgtaagg
361 aaataactga taaggaattg gacaagaaaa tgaaggagct cgccgccgtc
atgagcgacc
421 ctgacctgga aactgagact atgtgcctcc acgacgacga gtcgtgtcgc
tacgaagggc

481 aagtcgctgt ttaccaggat gtatacgcgg ttgacggacc gacaagtctc tatcaccaag
541 ccaataaggg agttagagtc gcctactgga taggctttga caccacccct tttatgttta
601 agaacttggc tggagcatat ccatcatact ctaccaactg ggccgacgaa accgtgttaa
661 cggctcgtaa cataggccta tgcagctctg acgttatgga gcggtcacgt agagggatgt
721 ccattcttag aaagaagtat ttgaaaccat ccaacaatgt tctattctct gttggctcga
781 ccatctacca cgagaagagg gacttactga ggagctggca cctgccgtct gtatttcact
841 tacgtggcaa gcaaaattac acatgtcggt gtgagactat agttagttgc gacgggtacg
901 tcgttaaaag aatagctatc agtccaggcc tgtatgggaa gccttcaggc tatgctgcta
961 cgatgcaccg cgagggattc ttgtgctgca aagtgacaga cacattgaac ggggagaggg
1021 tctcttttcc cgtgtgcacg tatgtgccag ctacattgtg tgaccaaatg actggcatac
1081 tggcaacaga tgtcagtgcg gacgacgcgc aaaaactgct ggttgggctc aaccagcgta
1141 tagtcgtcaa cggtcgcacc cagagaaaca ccaataccat gaaaaattac cttttgcccg
1201 tagtggccca ggcatttgct aggtgggcaa aggaatataa ggaagatcaa gaagatgaaa
1261 ggccactagg actacgagat agacagttag tcatggggtg ttgttgggct tttagaaggc
1321 acaagataac atctatttat aagcgcccgg atacccaaac catcatcaaa gtgaacagcg
1381 atttccactc attcgtgctg cccaggatag gcagtaacac attggagatc gggctgagaa
1441 caagaatcag gaaaatgtta gaggagcaca aggagccgtc acctctcatt accgccgagg
1501 acgtacaaga agctaagtgc gcagccgatg aggctaagga ggtgcgtgaa gccgaggagt
1561 tgcgcgcagc tctaccacct ttggcagctg atgttgagga gcccactctg gaagccgatg
1621 tcgacttgat gttacaagag gctggggccg gctcagtgga gacacctcgt ggcttgataa
1681 aggttaccag ctacgatggc gaggacaaga tcggctctta cgctgtgctt tctccgcagg
1741 ctgtactcaa gagtgaaaaa ttatcttgca tccaccctct cgctgaacaa gtcatagtga
1801 taacacactc tggccgaaaa gggcgttatg ccgtggaacc ataccatggt aaagtagtgg
1861 tgccagaggg acatgcaata cccgtccagg actttcaagc tctgagtgaa agtgccacca
1921 ttgtgtacaa cgaacgtgag ttcgtaaaca ggtacctgca ccatattgcc acacatggag
1981 gagcgctgaa cactgatgaa gaatattaca aaactgtcaa gcccagcgag cacgacggcg

2041 aatacctgta cgacatcgac aggaaacagt gcgtcaagaa agaactagtc actgggctag
2101 ggctcacagg cgagctggtg gatcctccct tccatgaatt cgcctacgag agtctgagaa
2161 cacgaccagc cgctccttac caagtaccaa ccataggggt gtatggcgtg ccaggatcag
2221 gcaagtctgg catcattaaa agcgcagtca ccaaaaaaga tctagtggtg agcgccaaga
2281 aagaaaactg tgcagaaatt ataagggacg tcaagaaaat gaaagggctg gacgtcaatg
2341 ccagaactgt ggactcagtg ctcttgaatg gatgcaaaca ccccgtagag accctgtata
2401 ttgacgaagc ttttgcttgt catgcaggta ctctcagagc gctcatagcc attataagac
2461 ctaaaaaggc agtgctctgc ggggatccca aacagtgcgg tttttttaac atgatgtgcc
2521 tgaaagtgca ttttaaccac gagatttgca cacaagtctt ccacaaaagc atctctcgcc
2581 gttgcactaa atctgtgact tcggtcgtct caaccttgtt ttacgacaaa aaaatgagaa
2641 cgacgaatcc gaaagagact aagattgtga ttgacactac cggcagtacc aaacctaagc
2701 aggacgatct cattctcact tgtttcagag ggtgggtgaa gcagttgcaa atagattaca
2761 aaggcaacga aataatgacg gcagctgcct ctcaagggct gacccgtaaa ggtgtgtatg
2821 ccgttcggta caaggtgaat gaaaatcctc tgtacgcacc cacctcagaa catgtgaacg
2881 tcctactgac ccgcacggag gaccgcatcg tgtggaaaac actagccggc gacccatgga
2941 taaaaacact gactgccaag taccctggga atttcactgc cacgatagag gagtggcaag
3001 cagagcatga tgccatcatg aggcacatct tggagagacc ggaccctacc gacgtcttcc
3061 agaataaggc aaacgtgtgt tgggccaagg ctttagtgcc ggtgctgaag accgctggca
3121 tagacatgac cactgaacaa tggaacactg tggattattt tgaaacggac aaagctcact
3181 cagcagagat agtattgaac caactatgcg tgaggttctt tggactcgat ctggactccg
3241 gtctattttc tgcacccact gttccgttat ccattaggaa taatcactgg gataactccc
3301 cgtcgcctaa catgtacggg ctgaataaag aagtggtccg tcagctctct cgcaggtacc
3361 cacaactgcc tcgggcagtt gccactggaa gagtctatga catgaacact ggtacactgc
3421 gcaattatga tccgcgcata aacctagtac ctgtaaacag aagactgcct catgctttag
3481 tcctccacca taatgaacac ccacagagtg acttttcttc attcgtcagc aaattgaagg
3541 gcagaactgt cctggtggtc ggggaaaagt tgtccgtccc aggcaaaatg gttgactggt

3601 tgtcagaccg gcctgaggct accttcagag ctcggctgga tttaggcatc ccaggtgatg
3661 tgcccaaata tgacataata tttgttaatg tgaggacccc atataaatac catcactatc
3721 agcagtgtga agaccatgcc attaagctta gcatgttgac caagaaagct tgtctgcatc
3781 tgaatcccgg cggaacctgt gtcagcatag gttatggtta cgctgacagg gccagcgaaa
3841 gcatcattgg tgctatagcg cggcagttca agttttcccg ggtatgcaaa ccgaaatcct
3901 cacttgaaga gacggaagtt ctgtttgtat tcattgggta cgatcgcaag gcccgtacgc
3961 acaatcctta caagctttca tcaaccttga ccaacattta tacaggttcc agactccacg
4021 aagccggatg tgcaccctca tatcatgtgg tgcgagggga tattgccacg gccaccgaag
4081 gagtgattat aaatgctgct aacagcaaag gacaacctgg cggaggggtg tgcggagcgc
4141 tgtataagaa attcccggaa agcttcgatt tacagccgat cgaagtagga aaagcgcgac
4201 tggtcaaagg tgcagctaaa catatcattc atgccgtagg accaaacttc aacaaagttt
4261 cggaggttga aggtgacaaa cagttggcag aggcttatga gtccatcgct aagattgtca
4321 acgataacaa ttacaagtca gtagcgattc cactgttgtc caccggcatc ttttccggga
4381 acaaagatcg actaacccaa tcattgaacc atttgctgac agctttagac accactgatg
4441 cagatgtagc catatactgc agggacaaga aatgggaaat gactctcaag gaagcagtgg
4501 ctaggagaga agcagtggag gagatatgca tatccgacga ctcttcagtg acagaacctg
4561 atgcagagct ggtgagggtg catccgaaga gttctttggc tggaaggaag ggctacagca
4621 caagcgatgg caaaactttc tcatatttgg aagggaccaa gtttcaccag gcggccaagg
4681 atatagcaga aattaatgcc atgtggcccg ttgcaacgga ggccaatgag caggtatgca
4741 tgtatatcct cggagaaagc atgagcagta ttaggtcgaa atgccccgtc gaagagtcgg
4801 aagcctccac accacctagc acgctgcctt gcttgtgcat ccatgccatg actccagaaa
4861 gagtacagcg cctaaaagcc tcacgtccag aacaaattac tgtgtgctca tcctttccat
4921 tgccgaagta tagaatcact ggtgtgcaga agatccaatg ctcccagcct atattgttct
4981 caccgaaagt gcctgcgtat attcatccaa ggaagtatct cgtggaaaca ccaccggtag
5041 acgagactcc ggagccatcg gcagagaacc aatccacaga ggggacacct gaacaaccac
5101 cacttataac cgaggatgag accaggacta gaacgcctga gccgatcatc atcgaagagg

5161 aagaagagga tagcataagt ttgctgtcag atggcccgac ccaccaggtg ctgcaagtcg
5221 aggcagacat tcacgggccg ccctctgtat ctagctcatc ctggtccatt cctcatgcat
5281 ccgactttga tgtggacagt ttatccatac ttgacaccct ggagggagct agcgtgacca
5341 gcggggcaac gtcagccgag actaactctt acttcgcaaa gagtatggag tttctggcgc
5401 gaccggtgcc tgcgcctcga acagtattca ggaaccctcc acatcccgct ccgcgcacaa
5461 gaacaccgtc acttgcaccc agcagggcct gctcgagaac cagcctagtt tccaccccgc
5521 caggcgtgaa tagggtgatc actagagagg agctcgaggc gcttaccccg tcacgcactc
5581 ctagcaggtc ggtctcgaga accagcctgg tctccaaccc gccaggcgta aatagggtga
5641 ttacaagaga ggagtttgag gcgttcgtag cacaacaaca atgacggttt gatgcgggtg
5701 catacatctt ttcctccgac accggtcaag ggcatttaca acaaaaatca gtaaggcaaa
5761 cggtgctatc cgaagtggtg ttggagagga ccgaattgga gatttcgtat gccccgcgcc
5821 tcgaccaaga aaaagaagaa ttactacgca agaaattaca gttaaatccc acacctgcta
5881 acagaagcag ataccagtcc aggaaggtgg agaacatgaa agccataaca gctagacgta
5941 ttctgcaagg cctagggcat tatttgaagg cagaaggaaa agtggagtgc taccgaaccc
6001 tgcatcctgt tcctttgtat tcatctagtg tgaaccgtgc cttttcaagc cccaaggtcg
6061 cagtggaagc ctgtaacgcc atgttgaaag agaactttcc gactgtggct tcttactgta
6121 ttattccaga gtacgatgcc tatttggaca tggttgacgg agcttcatgc tgcttagaca
6181 ctgccagttt ttgccctgca aagctgcgca gctttccaaa gaaacactcc tatttggaac
6241 ccacaatacg atcggcagtg ccttcagcga tccagaacac gctccagaac gtcctggcag
6301 ctgccacaaa aagaaattgc aatgtcacgc aaatgagaga attgcccgta ttggattcgg
6361 cggcctttaa tgtggaatgc ttcaagaaat atgcgtgtaa taatgaatat tgggaaacgt
6421 ttaaagaaaa ccccatcagg cttactgaag aaaacgtggt aaattacatt accaaattaa
6481 aaggaccaaa agctgctgct ctttttgcga agacacataa tttgaatatg ttgcaggaca
6541 taccaatgga caggtttgta atggacttaa agagagacgt gaaagtgact ccaggaacaa
6601 aacatactga agaacggccc aaggtacagg tgatccaggc tgccgatccg ctagcaacag
6661 cgtatctgtg cggaatccac cgagagctgg ttaggagatt aaatgcggtc ctgcttccga

6721 acattcatac actgtttgat atgtcggctg aagactttga cgctattata gccgagcact
6781 tccagcctgg ggattgtgtt ctggaaactg acatcgcgtc gtttgataaa agtgaggacg
6841 acgccatggc tctgaccgcg ttaatgattc tggaagactt aggtgtggac gcagagctgt
6901 tgacgctgat tgaggcggct ttcggcgaaa tttcatcaat acatttgccc actaaaacta
6961 aatttaaatt cggagccatg atgaaatctg gaatgttcct cacactgttt gtgaacacag
7021 tcattaacat tgtaatcgca agcagagtgt tgagagaacg gctaaccgga tcaccatgtg
7081 cagcattcat tggagatgac aatatcgtga aaggagtcaa atcggacaaa ttaatggcag
7141 acaggtgcgc cacctggttg aatatggaag tcaagattat agatgctgtg gtgggcgaga
7201 aagcgcctta tttctgtgga gggtttattt tgtgtgactc cgtgaccggc acagcgtgcc
7261 gtgtggcaga cccccctaaaa aggctgttta agcttggcaa acctctggca gcagacgatg
7321 aacatgatga tgacaggaga agggcattgc atgaagagtc aacacgctgg aaccgagtgg
7381 gtattctttc agagctgtgc aaggcagtag aatcaaggta tgaaaccgta ggaacttcca
7441 tcatagttat ggccatgact actctagcta gcagtgttaa atcattcagc tacctgagag
7501 gggcccctat aactctctac ggctaacctg aatggactac gacatagtct agtccgccaa
7561 gatgttcccg ttccagccaa tgtatccgat gcagccaatg ccctatcgca acccgttcgc
7621 ggccccgcgc aggccctggt tccccagaac cgaccctttt ctggcgatgc aggtgcagga
7681 attaacccgc tcgatggcta acctgacgtt caagcaacgc cgggacgcgc cacctgaggg
7741 gccatccgct aagaaaccga agaaggaggc ctcgcaaaaa cagaaagggg gaggccaagg
7801 gaagaagaag aagaaccaag ggaagaagaa ggctaagaca gggccgccta atccgaaggc
7861 acagaatgga aacaagaaga agaccaacaa gaaaccaggc aagagacagc gcatggtcat
7921 gaaattggaa tctgacaaga cgttcccaat catgttggaa gggaagataa acggctacgc
7981 ttgtgtggtc ggagggaagt tattcaggcc gatgcatgtg gaaggcaaga tcgacaacga
8041 cgttctggcc gcgcttaaga cgaagaaagc atccaaatac gatcttgagt atgcagatgt
8101 gccacagaac atgcgggccg atacattcaa atacacccat gagaaacccc aaggctatta
8161 cagctggcat catggagcag tccaatatga aaatgggcgt ttcacggtgc cgaaaggagt
8221 tggggccaag ggagacagcg gacgacccat tctggataac cagggacggg tggtcgctat

8281 tgtgctggga ggtgtgaatg aaggatctag gacagccctt tcagtcgtca tgtggaacga
8341 gaagggagtt accgtgaagt atactccgga gaactgcgag caatggtcac tagtgaccac
8401 catgtgtctg ctcgccaatg tgacgttccc atgtgctcaa ccaccaattt gctacgacag
8461 aaaaccagca gagactttgg ccatgctcag cgttaacgtt gacaacccgg gctacgatga
8521 gctgctggaa gcagctgtta agtgccccgg aaggaaaagg agatccaccg aggagctgtt
8581 taatgagtat aagctaacgc gcccttacat ggccagatgc atcagatgtg cagttgggag
8641 ctgccatagt ccaatagcaa tcgaggcagt aaagagcgac gggcacgacg gttatgttag
8701 acttcagact tcctcgcagt atggcctgga ttcctccggc aacttaaagg gcaggaccat
8761 gcggtatgac atgcacggga ccattaaaga gataccacta catcaagtgt cactctatac
8821 atctcgcccg tgtcacattg tggatgggca cggttatttc ctgcttgcca ggtgcccggc
8881 aggggactcc atcaccatgg aatttaagaa agattccgtc acacactcct gctcggtgcc
8941 gtatgaagtg aaatttaatc ctgtaggcag agaactctat actcatcccc cagaacacgg
9001 agtagagcaa gcgtgccaag tctacgcaca tgatgcacag aacagaggag cttatgtcga
9061 gatgcacctc ccgggctcag aagtggacag cagtttggtt tccttgagcg gcagttcagt
9121 caccgtgaca cctcctgatg ggactagcgc cctggtggaa tgcgagtgtg gcggcacaaa
9181 gatctccgag accatcaaca agacaaaaca gttcagccag tgcacaaaga aggagcagtg
9241 cagagcatat cggctgcaga acgataagtg ggtgtataat tctgacaaac tgcccaaagc
9301 agcgggagcc accttaaaag gaaaactgca tgtcccattc ttgctggcag acggcaaatg
9361 caccgtgcct ctagcaccag aacctatgat aaccttcggt ttcagatcag tgtcactgaa
9421 actgcaccct aagaatccca catatctaat cacccgccaa cttgctgatg agcctcacta
9481 cacgcacgag ctcatatctg aaccagctgt taggaatttt accgtcaccg aaaaagggtg
9541 ggagtttgta tggggaaacc acccgccgaa aaggttttgg gcacaggaaa cagcacccgg
9601 aaatccacat gggctaccgc acgaggtgat aactcattat taccacagat accctatgtc
9661 caccatcctg ggtttgtcaa tttgtgccgc cattgcaacc gtttccgttg cagcgtctac
9721 ctggctgttt tgcagatcta gagttgcgtg cctaactcct taccggctaa cacctaacgc
9781 taggatacca ttttgtctgg ctgtgctttg ctgcgcccgc actgcccggg ccgagaccac

9841 ctgggagtcc ttggatcacc tatggaacaa taaccaacag atgttctgga ttcaattgct
9901 gatccctctg gccgccttga tcgtagtgac tcgcctgctc aggtgcgtgt gctgtgtcgt
9961 gccttttta gtcatggccg gcgccgcagg cgccggcgcc tacgagcacg cgaccacgat
10021 gccgagccaa gcgggaatct cgtataacac tatagtcaac agagcaggct acgcaccact
10081 ccctatcagc ataacaccaa caaagatcaa gctgatacct acagtgaact tggagtacgt
10141 cacctgccac tacaaaacag gaatggattc accagccatc aaatgctgcg gatctcagga
10201 atgcactcca acttacaggc ctgatgaaca gtgcaaagtc ttcacagggg tttacccgtt
10261 catgtggggt ggtgcatatt gcttttgcga cactgagaac acccaagtca gcaaggccta
10321 cgtaatgaaa tctgacgact gccttgcgga tcatgctgaa gcatataaag cgcacacagc
10381 ctcagtgcag gcgttcctca acatcacagt gggagaacac tctattgtga ctaccgtgta
10441 tgtgaatgga gaaactcctg tgaatttcaa tggggtcaaa ataactgcag gtccgctttc
10501 cacagcttgg acaccctttg atcgcaaaat cgtgcagtat gccggggaga tctataatta
10561 tgattttcct gagtatgggg caggacaacc aggagcattt ggagatatac aatccagaac
10621 agtctcaagc tctgatctgt atgccaatac caacctagtg ctgcagagac ccaaagcagg
10681 agcgatccac gtgccataca ctcaggcacc ttcgggtttt gagcaatgga agaaagataa
10741 agctccatca ttgaaattta ccgccccttt cggatgcgaa atatatacaa accccattcg
10801 cgccgaaaac tgtgctgtag ggtcaattcc attagccttt gacattcccg acgccttgtt
10861 caccagggtg tcagaaacac cgacactttc agcggccgaa tgcactctta acgagtgcgt
10921 gtattcttcc gactttggtg ggatcgccac ggtcaagtac tcggccagca agtcaggcaa
10981 gtgcgcagtc catgtgccat cagggactgc taccctaaaa gaagcagcag tcgagctaac
11041 cgagcaaggg tcggcgacta tccatttctc gaccgcaaat atccacccgg agttcaggct
11101 ccaaatatgc acatcatatg ttacgtgcaa aggtgattgt caccccccga aagaccatat
11161 tgtgacacac cctcagtatc acgcccaaac atttacagcc gcggtgtcaa aaaccgcgtg
11221 gacgtggtta acatccctgc tgggaggatc agccgtaatt attataattg gcttggtgct
11281 ggctactatt gtggccatgt acgtgctgac caaccagaaa cataattgaa tacagcagca
11341 attggcaagc tgcttacata gaactcgcgg cgattggcat gccgccttaa aattttatt

11401 ttatttttct tttcttttcc gaatcggatt ttgtttttaa tatttcaaaa aaaaaaaaaa
11461 aaaaaaaaaa aagggtcggc atggcatctc cacctcctcg cggtccgacc tgggcatccg
11521 aaggaggacg cacgtccact cggatggcta agggagagcc acgagctcct cgacagatca
11581 taatcagcca taccacattt gtagaggttt tacttgcttt aaaaaacctc ccacacctcc
11641 ccctgaacct gaaacataaa atgaatgcaa ttgttgttgt taacttgttt attgcagctt
11701 ataatggtta caaataaagc aatagcatca caaatttcac aaataaagca tttttttcac
11761 tgcattctag ttgtggtttg tccaaactca tcaagatacg cgtacgcggc cccatgttcg
11821 ccttccgccg cgtggaggag gatcacagca acaccgagct gggcatcgtg gagtaccagc
11881 acgccttcaa gaccccggat gcagatgccg gtgaagaaag agtttaaacg gccggccgcg
11941 gtcatagctg tttcctgaac agatcccggg tggcatccct gtgacccctc cccagtgcct
12001 ctcctggccc tggaagttgc cactccagtg cccaccagcc ttgtcctaat aaaattaagt
12061 tgcatcattt tgtctgacta ggtgtccttc tataatatta tggggtggag gggggtggta
12121 tggagcaagg ggcaagttgg gaagacaacc tgtagggcct gcggggtcta ttgggaacca
12181 agctggagtg cagtggcaca atcttggctc actgcaatct ccgcctcctg ggttcaagcg
12241 attctcctgc ctcagcctcc cgagttgttg ggattccagg catgcatgac caggctcagc
12301 taatttttgt ttttttggta gagacggggt ttcaccatat tggccaggct ggtctccaac
12361 tcctaatctc aggtgatcta cccaccttgg cctcccaaat tgctgggatt acaggcgtga
12421 accactgctc ccttccctgt ccttctgatt ttaaaataac tataccagca ggaggacgtc
12481 cagacacagc ataggctacc tggccatgcc caaccggtgg gacatttgag ttgcttgctt
12541 ggcactgtcc tctcatgcgt tgggtccact cagtagatgc ctgttgaatt gggtacgcgg
12601 ccagcttggc tgtggaatgt gtgtcagtta gggtgtggaa agtccccagg ctccccagca
12661 ggcagaagta tgcaaagcat gcatctcaat tagtcagcaa ccaggtgtgg aaagtcccca
12721 ggctccccag caggcagaag tatgcaaagc atgcatctca attagtcagc aaccatagtc
12781 ccgcccctaa ctccgcccat cccgccccta actccgccca gttccgccca ttctccgccc
12841 catggctgac taattttttt tatttatgca gaggccgagg ccgcctcggc ctctgagcta
12901 ttccagaagt agtgaggagg cttttttgga ggcctaggct tttgcaaaaa gctcccggga

12961 gcttgtatat ccattttcgg atctgatcaa gagacaggat gaggatcgtt tcgcatgatt
13021 gaacaagatg gattgcacgc aggttctccg gccgcttggg tggagaggct attcggctat
13081 gactgggcac aacagacaat cggctgctct gatgccgccg tgttccggct gtcagcgcag
13141 gggcgcccgg ttctttttgt caagaccgac ctgtccggtg ccctgaatga actgcaggac
13201 gaggcagcgc ggctatcgtg gctggccacg acgggcgttc cttgcgcagc tgtgctcgac
13261 gttgtcactg aagcgggaag ggactggctg ctattgggcg aagtgccggg gcaggatctc
13321 ctgtcatctc accttgctcc tgccgagaaa gtatccatca tggctgatgc aatgcggcgg
13381 ctgcatacgc ttgatccggc tacctgccca ttcgaccacc aagcgaaaca tcgcatcgag
13441 cgagcacgta ctcggatgga agccggtctt gtcgatcagg atgatctgga cgaagagcat
13501 caggggctcg cgccagccga actgttcgcc aggctcaagg cgcgcatgcc cgacggcgag
13561 gatctcgtcg tgacccatgg cgatgcctgc ttgccgaata tcatggtgga aaatggccgc
13621 ttttctggat tcatcgactg tggccggctg ggtgtggccg accgctatca ggacatagcg
13681 ttggctaccc gtgatattgc tgaagagctt ggcggcgaat gggctgaccg cttcctcgtg
13741 ctttacggta tcgccgctcc cgattcgcag cgcatcgcct tctatcgcct tcttgacgag
13801 ttcttctgag cgggactctg gggttcgaaa tgaccgacca agcgacgccc aacctgccat
13861 cacgagattt cgattccacc gccgccttct atgaaaggtt gggcttcgga atcgttttcc
13921 gggacgccgg ctggatgatc ctccagcgcg gggatctcat gctggagttc ttcgcccacc
13981 ccaacttgtt tattgcagct tataatggtt acaaataaag caatagcatc acaaatttca
14041 caaataaagc atttttttca ctgcattcta gttgtggttt gtccaaactc atcaatgtat
14101 cttatcatgt ctgtatactg gcttactatg ttggcactga tgagggtgtc agtgaagtgc
14161 ttcatgtggc aggagaaaaa aggctgcacc ggtgcgtcag cagaatatgt gatacaggat
14221 atattccgct tcctcgctca ctgactcgct acgctcggtc gttcgactgc ggcgagcgga
14281 aatggcttac gaacggggcg gagatttcct ggaagatgcc aggaagatac ttaacaggga
14341 agtgagaggg ccgcggcaaa gccgtttttc cataggctcc gcccccctga caagcatcac
14401 gaaatctgac gctcaaatca gtggtggcga aacccgacag gactataaag ataccaggcg
14461 tttccccctgg cggctccctc gtgcgctctc ctgttcctgc ctttcggttt accggtgtca

14521 ttccgctgtt atggccgcgt ttgtctcatt ccacgcctga cactcagttc cgggtaggca
14581 gttcgctcca agctggactg tatgcacgaa ccccccgttc agtccgaccg ctgcgcctta
14641 tccggtaact atcgtcttga gtccaacccg gaaagacatg caaaagcacc actggcagca
14701 gccactggta attgatttag aggagttagt cttgaagtca tgcgccggtt aaggctaaac
14761 tgaaaggaca agttttggtg actgcgctcc tccaagccag ttacctcggt tcaaagagtt
14821 ggtagctcag agaaccttcg aaaaaccgcc ctgcaaggcg gttttttcgt tttcagagca
14881 agagattacg cgcagaccaa aacgatctca agaagatcat cttattaagg ggtctgacgc
14941 tcagtggaac gaaaactcac gttaagggat tttggtcatg agattatcaa aaaggatctt
15001 cacctagatc cttttaaatt aaaaatgaag ttttaaatca atctaaagta tatatgagta
15061 aacttggtct gacagttacc aatgcttaat cagtgaggca cctatctcag cgatctgtct
15121 atttcgttca tccatagttg cctgactccc cgtcgtgtag ataactacga tacgggaggg
15181 cttaccatct ggccccagtg ctgcaatgat accgcgagac ccacgctcac cggctccaga
15241 tttatcagca ataaaccagc cagccggaag ggccgagcgc agaagtggtc ctgcaacttt
15301 atccgcctcc atccagtcta ttaattgttg ccgggaagct agagtaagta gttcgccagt
15361 taatagtttg cgcaacgttg ttgccattgc tgcaggcatc gtggtgtcac gctcgtcgtt
15421 tggtatggct tcattcagct ccggttccca acgatcaagg cgagttacat gatcccccat
15481 gttgtgcaaa aaagcggtta gctccttcgg tcctccgatc gttgtcagaa gtaagttggc
15541 cgcagtgtta tcactcatgg ttatggcagc actgcataat tctcttactg tcatgccatc
15601 cgtaagatgc ttttctgtga ctggtgagta ctcaaccaag tcattctgag aatagtgtat
15661 gcggcgaccg agttgctctt gcccggcgtc aacacgggat aataccgcgc cacatagcag
15721 aactttaaaa gtgctcatca ttggaaaacg ttcttcgggg cgaaaactct caaggatctt
15781 accgctgttg agatccagtt cgatgtaacc cactcgtgca cccaactgat cttcagcatc
15841 ttttactttc accagcgttt ctgggtgagc aaaaacagga aggcaaaatg ccgcaaaaaa
15901 gggaataagg gcgacacgga aatgttgaat actcatactc ttcctttttc aatattattg
15961 aagcatttat cagggttatt gtctcatgag cggatacata tttgaatgta tttagaaaaa
16021 taaacaaata ggggttccgc gcacatttcc ccgaaaagtg ccacctgacg tgtcgacgcg

16081 gccgcacatt gattattgac tagttattaa tagtaatcaa ttacggggtc attagttcat
16141 agcccatata tggagttccg cgttacataa cttacggtaa atggcccgcc tggctgaccg
16201 cccaacgacc cccgcccatt gacgtcaata atgacgtatg ttcccatagt aacgccaata
16261 gggactttcc attgacgtca atgggtggag tatttacggt aaactgccca cttggcagta
16321 catcaagtgt atcatatgcc aagtccgccc cctattgacg tcaatgacgg taaatggccc
16381 gcctggcatt atgcccagta catgacctta cgggactttc ctacttggca gtacatctac
16441 gtattagtca tcgctattac catggtgatg cggttttggc agtacaccaa tgggcgtgga
16501 tagcggtttg actcacgggg atttccaagt ctccacccca ttgacgtcaa tgggagtttg
16561 ttttggcacc aaaatcaacg ggactttcca aaatgtcgta ataaccccgc cccgttgacg
16621 caaatgggcg gtaggcgtgt acggtgggag gtctatataa gcagagctcg tttagtgaac
16681 cg
//

Figure S7. Nucleotide sequence of the cTC-83/TrD-GFP molecular infectious clone

```
LOCUS       cTC-83_TrD-GFP       17540 bp    DNA     circular     10-JAN-2022
SOURCE
  ORGANISM
FEATURES             Location/Qualifiers
     mutation        9780..9780
                     /vntifkey="62"
                     /label=G->C\(Arg->Tre)\(TrD)
     mutation        3..3
                     /vntifkey="62"
                     /label=A->G\(TrD)
     CDS             13873..14664
                     /vntifkey="4"
                     /label=Neo
     CDS             10859..12184
                     /vntifkey="4"
                     /label=E1
     CDS             8420..9244
                     /vntifkey="4"
                     /label=C
     5'UTR           1..44
                     /vntifkey="52"
                     /label=5'UTR
     CDS             45..1649
                     /product="nsP1"
                     /vntifkey="4"
                     /label=nsP1
                     /note="putative"
     CDS             1650..4031
                     /product="nsP2"
                     /function="replication"
                     /vntifkey="4"
                     /label=nsP2
                     /note="putative"
     CDS             4032..5702
                     /product="nsP3"
                     /function="replication"
                     /vntifkey="4"
                     /label=nsP3
                     /note="putative"
     CDS             9245..9421
                     /product="E3"
                     /function="incorporated into the envelope of some alpha
                     viruses"
                     /vntifkey="4"
                     /label=E3
                     /note="putative"
     CDS             10691..10858
                     /product="6K protein"
                     /vntifkey="4"
                     /label=6k
```

```
                     /note="putative"
     CDS             9422..10690
                     /vntifkey="4"
                     /label=E2
     misc_feature    12823..4
                     /vntifkey="21"
                     /label=plasmid\vector
     misc_RNA        12331..12414
                     /vntifkey="53"
                     /label=Antigenomic\RBZ\HDV
     promoter        16969..17540
                     /vntifkey="29"
                     /label=CMV\promoter
     misc_feature    13493..13795
                     /vntifkey="21"
                     /label=SV40\ori
     polyA_signal    12823..13408
                     /vntifkey="25"
                     /label=PolyA\signal\from\HGH\(human\growth\hormone)
     CDS             complement(15934..16794)
                     /vntifkey="4"
                     /label=Ap
     rep_origin      15224..15769
                     /vntifkey="33"
                     /label=p15A\origin\of\replication
     CDS             7581..8297
                     /vntifkey="4"
                     /label=GFP
     CDS             5703..7523
                     /vntifkey="4"
                     /label=nsP4
     promoter        7501..7545
                     /vntifkey="29"
                     /label=SP1
     promoter        8359..8403
                     /vntifkey="29"
                     /label=SP2
BASE COUNT     4692 a     4447 c     4443 g     3958 t
ORIGIN
        1 atgggcggcg catgagagaa gcccagacca attacctacc caaaatggag
aaagttcacg
       61 ttgacatcga ggaagacagc ccattcctca gagctttgca gcggagcttc
ccgcagtttg
      121 aggtagaagc caagcaggtc actgataatg accatgctaa tgccagagcg
ttttcgcatc
      181 tggcttcaaa actgatcgaa acggaggtgg acccatccga cacgatcctt
gacattggaa
      241 gtgcgcccgc ccgcagaatg tattctaagc acaagtatca ttgtatctgt
ccgatgagat
      301 gtgcggaaga tccggacaga ttgtataagt atgcaactaa gctgaagaaa
aactgtaagg
      361 aaataactga taaggaattg gacaagaaaa tgaaggagct cgccgccgtc
atgagcgacc
```

421 ctgacctgga aactgagact atgtgcctcc acgacgacga gtcgtgtcgc tacgaagggc
481 aagtcgctgt ttaccaggat gtatacgcgg ttgacggacc gacaagtctc tatcaccaag
541 ccaataaggg agttagagtc gcctactgga taggctttga caccacccct tttatgttta
601 agaacttggc tggagcatat ccatcatact ctaccaactg ggccgacgaa accgtgttaa
661 cggctcgtaa cataggccta tgcagctctg acgttatgga gcggtcacgt agagggatgt
721 ccattcttag aaagaagtat ttgaaaccat ccaacaatgt tctattctct gttggctcga
781 ccatctacca cgagaagagg gacttactga ggagctggca cctgccgtct gtatttcact
841 tacgtggcaa gcaaaattac acatgtcggt gtgagactat agttagttgc gacgggtacg
901 tcgttaaaag aatagctatc agtccaggcc tgtatgggaa gccttcaggc tatgctgcta
961 cgatgcaccg cgagggattc ttgtgctgca aagtgacaga cacattgaac ggggagaggg
1021 tctcttttcc cgtgtgcacg tatgtgccag ctacattgtg tgaccaaatg actggcatac
1081 tggcaacaga tgtcagtgcg gacgacgcgc aaaaactgct ggttgggctc aaccagcgta
1141 tagtcgtcaa cggtcgcacc cagagaaaca ccaataccat gaaaaattac cttttgcccg
1201 tagtggccca ggcatttgct aggtgggcaa aggaatataa ggaagatcaa gaagatgaaa
1261 ggccactagg actacgagat agacagttag tcatggggtg ttgttgggct tttagaaggc
1321 acaagataac atctatttat aagcgcccgg atacccaaac catcatcaaa gtgaacagcg
1381 atttccactc attcgtgctg cccaggatag gcagtaacac attggagatc gggctgagaa
1441 caagaatcag gaaaatgtta gaggagcaca aggagccgtc acctctcatt accgccgagg
1501 acgtacaaga agctaagtgc gcagccgatg aggctaagga ggtgcgtgaa gccgaggagt
1561 tgcgcgcagc tctaccacct ttggcagctg atgttgagga gcccactctg gaagccgatg
1621 tcgacttgat gttacaagag gctggggccg gctcagtgga gacacctcgt ggcttgataa
1681 aggttaccag ctacgatggc gaggacaaga tcggctctta cgctgtgctt tctccgcagg
1741 ctgtactcaa gagtgaaaaa ttatcttgca tccaccctct cgctgaacaa gtcatagtga
1801 taacacactc tggccgaaaa gggcgttatg ccgtggaacc ataccatggt aaagtagtgg
1861 tgccagaggg acatgcaata cccgtccagg actttcaagc tctgagtgaa agtgccacca
1921 ttgtgtacaa cgaacgtgag ttcgtaaaca ggtacctgca ccatattgcc acacatggag

1981 gagcgctgaa cactgatgaa gaatattaca aaactgtcaa gcccagcgag cacgacggcg
2041 aatacctgta cgacatcgac aggaaacagt gcgtcaagaa agaactagtc actgggctag
2101 ggctcacagg cgagctggtg gatcctccct tccatgaatt cgcctacgag agtctgagaa
2161 cacgaccagc cgctccttac caagtaccaa ccataggggt gtatggcgtg ccaggatcag
2221 gcaagtctgg catcattaaa agcgcagtca ccaaaaaaga tctagtggtg agcgccaaga
2281 aagaaaactg tgcagaaatt ataaggacg tcaagaaaat gaaagggctg gacgtcaatg
2341 ccagaactgt ggactcagtg ctcttgaatg gatgcaaaca ccccgtagag accctgtata
2401 ttgacgaagc ttttgcttgt catgcaggta ctctcagagc gctcatagcc attataagac
2461 ctaaaaaggc agtgctctgc ggggatccca aacagtgcgg tttttttaac atgatgtgcc
2521 tgaaagtgca ttttaaccac gagatttgca cacaagtctt ccacaaaagc atctctcgcc
2581 gttgcactaa atctgtgact tcggtcgtct caaccttgtt ttacgacaaa aaaatgagaa
2641 cgacgaatcc gaaagagact aagattgtga ttgacactac cggcagtacc aaacctaagc
2701 aggacgatct cattctcact tgtttcagag ggtgggtgaa gcagttgcaa atagattaca
2761 aaggcaacga aataatgacg gcagctgcct ctcaagggct gacccgtaaa ggtgtgtatg
2821 ccgttcggta caaggtgaat gaaaatcctc tgtacgcacc cacctcagaa catgtgaacg
2881 tcctactgac ccgcacggag gaccgcatcg tgtggaaaac actagccggc gacccatgga
2941 taaaaacact gactgccaag taccctggga atttcactgc cacgatagag gagtggcaag
3001 cagagcatga tgccatcatg aggcacatct tggagagacc ggaccctacc gacgtcttcc
3061 agaataaggc aaacgtgtgt tgggccaagg ctttagtgcc ggtgctgaag accgctggca
3121 tagacatgac cactgaacaa tggaacactg tggattattt tgaaacggac aaagctcact
3181 cagcagagat agtattgaac caactatgcg tgaggttctt tggactcgat ctggactccg
3241 gtctattttc tgcacccact gttccgttat ccattaggaa taatcactgg gataactccc
3301 cgtcgcctaa catgtacggg ctgaataaag aagtggtccg tcagctctct cgcaggtacc
3361 cacaactgcc tcgggcagtt gccactggaa gagtctatga catgaacact ggtacactgc
3421 gcaattatga tccgcgcata aacctagtac ctgtaaacag aagactgcct catgctttag
3481 tcctccacca taatgaacac ccacagagtg acttttcttc attcgtcagc aaattgaagg

3541 gcagaactgt cctggtggtc ggggaaaagt tgtccgtccc aggcaaaatg gttgactggt
3601 tgtcagaccg gcctgaggct accttcagag ctcggctgga tttaggcatc ccaggtgatg
3661 tgcccaaata tgacataata tttgttaatg tgaggacccc atataaatac catcactatc
3721 agcagtgtga agaccatgcc attaagctta gcatgttgac caagaaagct tgtctgcatc
3781 tgaatcccgg cggaacctgt gtcagcatag gttatggtta cgctgacagg gccagcgaaa
3841 gcatcattgg tgctatagcg cggcagttca agttttcccg ggtatgcaaa ccgaaatcct
3901 cacttgaaga gacggaagtt ctgtttgtat tcattgggta cgatcgcaag gcccgtacgc
3961 acaatcctta caagctttca tcaaccttga ccaacattta tacaggttcc agactccacg
4021 aagccggatg tgcaccctca tatcatgtgg tgcgagggga tattgccacg gccaccgaag
4081 gagtgattat aaatgctgct aacagcaaag gacaacctgg cggaggggtg tgcggagcgc
4141 tgtataagaa attcccggaa agcttcgatt tacagccgat cgaagtagga aaagcgcgac
4201 tggtcaaagg tgcagctaaa catatcattc atgccgtagg accaaacttc aacaaagttt
4261 cggaggttga aggtgacaaa cagttggcag aggcttatga gtccatcgct aagattgtca
4321 acgataacaa ttacaagtca gtagcgattc cactgttgtc caccggcatc ttttccggga
4381 acaaagatcg actaacccaa tcattgaacc atttgctgac agctttagac accactgatg
4441 cagatgtagc catatactgc agggacaaga aatgggaaat gactctcaag gaagcagtgg
4501 ctaggagaga agcagtggag gagatatgca tatccgacga ctcttcagtg acagaacctg
4561 atgcagagct ggtgagggtg catccgaaga gttctttggc tggaaggaag ggctacagca
4621 caagcgatgg caaaactttc tcatatttgg aagggaccaa gtttcaccag gcggccaagg
4681 atatagcaga aattaatgcc atgtggcccg ttgcaacgga ggccaatgag caggtatgca
4741 tgtatatcct cggagaaagc atgagcagta ttaggtcgaa atgccccgtc gaagagtcgg
4801 aagcctccac accacctagc acgctgcctt gcttgtgcat ccatgccatg actccagaaa
4861 gagtacagcg cctaaaagcc tcacgtccag aacaaattac tgtgtgctca tcctttccat
4921 tgccgaagta tagaatcact ggtgtgcaga agatccaatg ctcccagcct atattgttct
4981 caccgaaagt gcctgcgtat attcatccaa ggaagtatct cgtggaaaca ccaccggtag
5041 acgagactcc ggagccatcg gcagagaacc aatccacaga ggggacacct gaacaaccac

5101 cacttataac cgaggatgag accaggacta gaacgcctga gccgatcatc atcgaagagg
5161 aagaagagga tagcataagt ttgctgtcag atggcccgac ccaccaggtg ctgcaagtcg
5221 aggcagacat tcacgggccg ccctctgtat ctagctcatc ctggtccatt cctcatgcat
5281 ccgactttga tgtggacagt ttatccatac ttgacaccct ggagggagct agcgtgacca
5341 gcggggcaac gtcagccgag actaactctt acttcgcaaa gagtatggag tttctggcgc
5401 gaccggtgcc tgcgcctcga acagtattca ggaaccctcc acatcccgct ccgcgcacaa
5461 gaacaccgtc acttgcaccc agcagggcct gctcgagaac cagcctagtt tccaccccgc
5521 caggcgtgaa tagggtgatc actagagagg agctcgaggc gcttaccccg tcacgcactc
5581 ctagcaggtc ggtctcgaga accagcctgg tctccaaccc gccaggcgta aatagggtga
5641 ttacaagaga ggagtttgag gcgttcgtag cacaacaaca atgacggttt gatgcgggtg
5701 catacatctt ttcctccgac accggtcaag ggcatttaca acaaaaatca gtaaggcaaa
5761 cggtgctatc cgaagtggtg ttggagagga ccgaattgga gatttcgtat gccccgcgcc
5821 tcgaccaaga aaaagaagaa ttactacgca agaaattaca gttaaatccc acacctgcta
5881 acagaagcag ataccagtcc aggaaggtgg agaacatgaa agccataaca gctagacgta
5941 ttctgcaagg cctagggcat tatttgaagg cagaaggaaa agtggagtgc taccgaaccc
6001 tgcatcctgt tcctttgtat tcatctagtg tgaaccgtgc cttttcaagc cccaaggtcg
6061 cagtggaagc ctgtaacgcc atgttgaaag agaactttcc gactgtggct tcttactgta
6121 ttattccaga gtacgatgcc tatttggaca tggttgacgg agcttcatgc tgcttagaca
6181 ctgccagttt ttgccctgca aagctgcgca gctttccaaa gaaacactcc tatttggaac
6241 ccacaatacg atcggcagtg ccttcagcga tccagaacac gctccagaac gtcctggcag
6301 ctgccacaaa aagaaattgc aatgtcacgc aaatgagaga attgcccgta ttggattcgg
6361 cggcctttaa tgtggaatgc ttcaagaaat atgcgtgtaa taatgaatat tgggaaacgt
6421 ttaaagaaaa ccccatcagg cttactgaag aaaacgtggt aaattacatt accaaattaa
6481 aaggaccaaa agctgctgct ctttttgcga agacacataa tttgaatatg ttgcaggaca
6541 taccaatgga caggtttgta atggacttaa agagagacgt gaaagtgact ccaggaacaa
6601 aacatactga agaacggccc aaggtacagg tgatccaggc tgccgatccg ctagcaacag

6661 cgtatctgtg cggaatccac cgagagctgg ttaggagatt aaatgcggtc ctgcttccga
6721 acattcatac actgtttgat atgtcggctg aagactttga cgctattata gccgagcact
6781 tccagcctgg ggattgtgtt ctggaaactg acatcgcgtc gtttgataaa agtgaggacg
6841 acgccatggc tctgaccgcg ttaatgattc tggaagactt aggtgtggac gcagagctgt
6901 tgacgctgat tgaggcggct ttcggcgaaa tttcatcaat acatttgccc actaaaacta
6961 aatttaaatt cggagccatg atgaaatctg gaatgttcct cacactgttt gtgaacacag
7021 tcattaacat tgtaatcgca agcagagtgt tgagagaacg gctaaccgga tcaccatgtg
7081 cagcattcat tggagatgac aatatcgtga aaggagtcaa atcggacaaa ttaatggcag
7141 acaggtgcgc cacctggttg aatatggaag tcaagattat agatgctgtg gtgggcgaga
7201 aagcgcctta tttctgtgga gggtttattt tgtgtgactc cgtgaccggc acagcgtgcc
7261 gtgtggcaga ccccctaaaa aggctgttta agcttggcaa acctctggca gcagacgatg
7321 aacatgatga tgacaggaga agggcattgc atgaagagtc aacacgctgg aaccgagtgg
7381 gtattctttc agagctgtgc aaggcagtag aatcaaggta tgaaaccgta ggaacttcca
7441 tcatagttat ggccatgact actctagcta gcagtgttaa atcattcagc tacctgagag
7501 gggcccctat aactctctac ggctaacctg aatggactac gacatagtct agtccgccaa
7561 gtctagagct tgccgccacc atggtgagca agggcgagga gctgttcacc ggggtggtgc
7621 ccatcctggt cgagctggac ggcgacgtaa acggccacaa gttcagcgtg tccggcgagg
7681 gcgagggcga tgccacctac ggcaagctga ccctgaagtt catctgcacc accggcaagc
7741 tgcccgtgcc ctggcccacc ctcgtgacca ccctgaccta cggcgtgcag tgcttcagcc
7801 gctaccccga ccacatgaag cagcacgact tcttcaagtc cgccatgccc gaaggctacg
7861 tccaggagcg caccatcttc ttcaaggacg acggcaacta caagacccgc gccgaggtga
7921 agttcgaggg cgacaccctg gtgaaccgca tcgagctgaa gggcatcgac ttcaaggagg
7981 acggcaacat cctggggcac aagctggagt acaactacaa cagccacaac gtctatatca
8041 tggccgacaa gcagaagaac ggcatcaagg tgaacttcaa gatccgccac aacatcgagg
8101 acggcagcgt gcagctcgcc gaccactacc agcagaacac ccccatcggc gacggccccg
8161 tgctgctgcc cgacaaccac tacctgagca cccagtccgc cctgagcaaa gaccccaacg

8221 agaagcgcga tcacatggtc ctgctggagt tcgtgaccgc cgccgggatc actctcggca
8281 tggacgagct gtacaagtaa agcggcccaa tgatccgacc agcaaaactc gatgtacttc
8341 cgaggaactg atgtgcataa gcccctataa ctctctacgg ctaacctgaa tggactacga
8401 catagtctag tccgccaaga tgttcccgtt ccagccaatg tatccgatgc agccaatgcc
8461 ctatcgcaac ccgttcgcgg ccccgcgcag gccctggttc cccagaaccg accctttct
8521 ggcgatgcag gtgcaggaat taacccgctc gatggctaac ctgacgttca agcaacgccg
8581 ggacgcgcca cctgaggggc catccgctaa gaaaccgaag aaggaggcct cgcaaaaaca
8641 gaaaggggga ggccaaggga agaagaagaa gaaccaaggg aagaagaagg ctaagacagg
8701 gccgcctaat ccgaaggcac agaatggaaa caagaagaag accaacaaga aaccaggcaa
8761 gagacagcgc atggtcatga aattggaatc tgacaagacg ttcccaatca tgttggaagg
8821 gaagataaac ggctacgctt gtgtggtcgg agggaagtta ttcaggccga tgcatgtgga
8881 aggcaagatc gacaacgacg ttctggccgc gcttaagacg aagaaagcat ccaaatacga
8941 tcttgagtat gcagatgtgc cacagaacat gcgggccgat acattcaaat acacccatga
9001 gaaaccccaa ggctattaca gctggcatca tggagcagtc caatatgaaa atgggcgttt
9061 cacggtgccg aaaggagttg gggccaaggg agacagcgga cgacccattc tggataacca
9121 gggacgggtg gtcgctattg tgctgggagg tgtgaatgaa ggatctagga cagccctttc
9181 agtcgtcatg tggaacgaga agggagttac cgtgaagtat actccggaga actgcgagca
9241 atggtcacta gtgaccacca tgtgtctgct cgccaatgtg acgttcccat gtgctcaacc
9301 accaatttgc tacgacagaa aaccagcaga gactttggcc atgctcagcg ttaacgttga
9361 caacccgggc tacgatgagc tgctggaagc agctgttaag tgccccggaa ggaaaaggag
9421 atccaccgag gagctgttta atgagtataa gctaacgcgc ccttacatgg ccagatgcat
9481 cagatgtgca gttgggagct gccatagtcc aatagcaatc gaggcagtaa agagcgacgg
9541 gcacgacggt tatgttagac ttcagacttc ctcgcagtat ggcctggatt cctccggcaa
9601 cttaaagggc aggaccatgc ggtatgacat gcacgggacc attaaagaga taccactaca
9661 tcaagtgtca ctctatacat ctcgcccgtg tcacattgtg gatgggcacg gttatttcct
9721 gcttgccagg tgcccggcag gggactccat caccatggaa tttaagaaag attccgtcac

9781 acactcctgc tcggtgccgt atgaagtgaa atttaatcct gtaggcagag aactctatac
9841 tcatccccca gaacacggag tagagcaagc gtgccaagtc tacgcacatg atgcacagaa
9901 cagaggagct tatgtcgaga tgcacctccc gggctcagaa gtggacagca gtttggtttc
9961 cttgagcggc agttcagtca ccgtgacacc tcctgatggg actagcgccc tggtggaatg
10021 cgagtgtggc ggcacaaaga tctccgagac catcaacaag acaaaacagt tcagccagtg
10081 cacaaagaag gagcagtgca gagcatatcg gctgcagaac gataagtggg tgtataattc
10141 tgacaaactg cccaaagcag cgggagccac cttaaaagga aaactgcatg tcccattctt
10201 gctggcagac ggcaaatgca ccgtgcctct agcaccagaa cctatgataa ccttcggttt
10261 cagatcagtg tcactgaaac tgcaccctaa gaatcccaca tatctaatca cccgccaact
10321 tgctgatgag cctcactaca cgcacgagct catatctgaa ccagctgtta ggaattttac
10381 cgtcaccgaa aaagggtggg agtttgtatg gggaaaccac ccgccgaaaa ggttttgggc
10441 acaggaaaca gcacccggaa atccacatgg gctaccgcac gaggtgataa ctcattatta
10501 ccacagatac cctatgtcca ccatcctggg tttgtcaatt tgtgccgcca ttgcaaccgt
10561 ttccgttgca gcgtctacct ggctgttttg cagatctaga gttgcgtgcc taactcctta
10621 ccggctaaca cctaacgcta ggataccatt ttgtctggct gtgctttgct gcgcccgcac
10681 tgcccgggcc gagaccacct gggagtcctt ggatcaccta tggaacaata accaacagat
10741 gttctggatt caattgctga tccctctggc cgccttgatc gtagtgactc gcctgctcag
10801 gtgcgtgtgc tgtgtcgtgc ctttttagt catggccggc gccgcaggcg ccggcgccta
10861 cgagcacgcg accacgatgc cgagccaagc gggaatctcg tataacacta tagtcaacag
10921 agcaggctac gcaccactcc ctatcagcat aacaccaaca aagatcaagc tgatacctac
10981 agtgaacttg gagtacgtca cctgccacta caaaacagga atggattcac cagccatcaa
11041 atgctgcgga tctcaggaat gcactccaac ttacaggcct gatgaacagt gcaaagtctt
11101 cacaggggtt tacccgttca tgtggggtgg tgcatattgc ttttgcgaca ctgagaacac
11161 ccaagtcagc aaggcctacg taatgaaatc tgacgactgc cttgcggatc atgctgaagc
11221 atataaagcg cacacagcct cagtgcaggc gttcctcaac atcacagtgg gagaacactc
11281 tattgtgact accgtgtatg tgaatggaga aactcctgtg aatttcaatg gggtcaaaat

11341 aactgcaggt ccgctttcca cagcttggac accctttgat cgcaaaatcg tgcagtatgc
11401 cggggagatc tataattatg attttcctga gtatggggca ggacaaccag gagcatttgg
11461 agatatacaa tccagaacag tctcaagctc tgatctgtat gccaatacca acctagtgct
11521 gcagagaccc aaagcaggag cgatccacgt gccatacact caggcacctt cgggttttga
11581 gcaatggaag aaagataaag ctccatcatt gaaatttacc gcccctttcg gatgcgaaat
11641 atatacaaac cccattcgcg ccgaaaactg tgctgtaggg tcaattccat tagcctttga
11701 cattcccgac gccttgttca ccagggtgtc agaaacaccg acactttcag cggccgaatg
11761 cactcttaac gagtgcgtgt attcttccga ctttggtggg atcgccacgg tcaagtactc
11821 ggccagcaag tcaggcaagt gcgcagtcca tgtgccatca gggactgcta ccctaaaaga
11881 agcagcagtc gagctaaccg agcaagggtc ggcgactatc catttctcga ccgcaaatat
11941 ccacccggag ttcaggctcc aaatatgcac atcatatgtt acgtgcaaag gtgattgtca
12001 ccccccgaaa gaccatattg tgacacaccc tcagtatcac gcccaaacat ttacagccgc
12061 ggtgtcaaaa accgcgtgga cgtggttaac atccctgctg ggaggatcag ccgtaattat
12121 tataattggc ttggtgctgg ctactattgt ggccatgtac gtgctgacca accagaaaca
12181 taattgaata cagcagcaat tggcaagctg cttacataga actcgcggcg attggcatgc
12241 cgccttaaaa tttttatttt attttctttt tcttttccga atcggatttt gtttttaata
12301 tttcaaaaaa aaaaaaaaaa aaaaaaaaaa gggtcggcat ggcatctcca cctcctcgcg
12361 gtccgacctg ggcatccgaa ggaggacgca cgtccactcg gatggctaag ggagagccac
12421 gagctcctcg acagatcata atcagccata ccacatttgt agaggtttta cttgctttaa
12481 aaaacctccc acacctcccc ctgaacctga aacataaaat gaatgcaatt gttgttgtta
12541 acttgtttat tgcagcttat aatggttaca aataaagcaa tagcatcaca aatttcacaa
12601 ataaagcatt tttttcactg cattctagtt gtggtttgtc caaactcatc aagatacgcg
12661 tacgcggccc catgttcgcc ttccgccgcg tggaggagga tcacagcaac accgagctgg
12721 gcatcgtgga gtaccagcac gccttcaaga ccccggatgc agatgccggt gaagaaagag
12781 tttaaacggc cggccgcggt catagctgtt tcctgaacag atcccgggtg gcatccctgt
12841 gacccctccc cagtgcctct cctggccctg gaagttgcca ctccagtgcc caccagcctt

12901 gtcctaataa aattaagttg catcattttg tctgactagg tgtccttcta taatattatg
12961 gggtggaggg gggtggtatg gagcaagggg caagttggga agacaacctg tagggcctgc
13021 ggggtctatt gggaaccaag ctggagtgca gtggcacaat cttggctcac tgcaatctcc
13081 gcctcctggg ttcaagcgat tctcctgcct cagcctcccg agttgttggg attccaggca
13141 tgcatgacca ggctcagcta atttttgttt ttttggtaga gacggggttt caccatattg
13201 gccaggctgg tctccaactc ctaatctcag gtgatctacc caccttggcc tcccaaattg
13261 ctgggattac aggcgtgaac cactgctccc ttccctgtcc ttctgatttt aaaataacta
13321 taccagcagg aggacgtcca gacacagcat aggctacctg gccatgccca accggtggga
13381 catttgagtt gcttgcttgg cactgtcctc tcatgcgttg ggtccactca gtagatgcct
13441 gttgaattgg gtacgcggcc agcttggctg tggaatgtgt gtcagttagg gtgtggaaag
13501 tccccaggct ccccagcagg cagaagtatg caaagcatgc atctcaatta gtcagcaacc
13561 aggtgtggaa agtccccagg ctccccagca ggcagaagta tgcaaagcat gcatctcaat
13621 tagtcagcaa ccatagtccc gcccctaact ccgcccatcc cgcccctaac tccgcccagt
13681 tccgcccatt ctccgcccca tggctgacta atttttttta tttatgcaga ggccgaggcc
13741 gcctcggcct ctgagctatt ccagaagtag tgaggaggct tttttggagg cctaggcttt
13801 tgcaaaaagc tcccgggagc ttgtatatcc attttcggat ctgatcaaga gacaggatga
13861 ggatcgtttc gcatgattga acaagatgga ttgcacgcag gttctccggc cgcttgggtg
13921 gagaggctat tcggctatga ctgggcacaa cagacaatcg gctgctctga tgccgccgtg
13981 ttccggctgt cagcgcaggg gcgcccggtt ctttttgtca agaccgacct gtccggtgcc
14041 ctgaatgaac tgcaggacga ggcagcgcgg ctatcgtggc tggccacgac gggcgttcct
14101 tgcgcagctg tgctcgacgt tgtcactgaa gcgggaaggg actggctgct attgggcgaa
14161 gtgccggggc aggatctcct gtcatctcac cttgctcctg ccgagaaagt atccatcatg
14221 gctgatgcaa tgcggcggct gcatacgctt gatccggcta cctgcccatt cgaccaccaa
14281 gcgaaacatc gcatcgagcg agcacgtact cggatggaag ccggtcttgt cgatcaggat
14341 gatctggacg aagagcatca ggggctcgcg ccagccgaac tgttcgccag gctcaaggcg
14401 cgcatgcccg acggcgagga tctcgtcgtg acccatggcg atgcctgctt gccgaatatc

14461 atggtggaaa atggccgctt ttctggattc atcgactgtg gccggctggg tgtggccgac
14521 cgctatcagg acatagcgtt ggctacccgt gatattgctg aagagcttgg cggcgaatgg
14581 gctgaccgct tcctcgtgct ttacggtatc gccgctcccg attcgcagcg catcgccttc
14641 tatcgccttc ttgacgagtt cttctgagcg ggactctggg gttcgaaatg accgaccaag
14701 cgacgcccaa cctgccatca cgagatttcg attccaccgc cgccttctat gaaaggttgg
14761 gcttcggaat cgttttccgg gacgccggct ggatgatcct ccagcgcggg gatctcatgc
14821 tggagttctt cgcccacccc aacttgttta ttgcagctta taatggttac aaataaagca
14881 atagcatcac aaatttcaca aataaagcat ttttttcact gcattctagt tgtggtttgt
14941 ccaaactcat caatgtatct tatcatgtct gtatactggc ttactatgtt ggcactgatg
15001 agggtgtcag tgaagtgctt catgtggcag gagaaaaaag gctgcaccgg tgcgtcagca
15061 gaatatgtga tacaggatat attccgcttc ctcgctcact gactcgctac gctcggtcgt
15121 tcgactgcgg cgagcggaaa tggcttacga acggggcgga gatttcctgg aagatgccag
15181 gaagatactt aacagggaag tgagagggcc gcggcaaagc cgtttttcca taggctccgc
15241 ccccctgaca agcatcacga aatctgacgc tcaaatcagt ggtggcgaaa cccgacagga
15301 ctataaagat accaggcgtt tccccctggcg gctccctcgt gcgctctcct gttcctgcct
15361 ttcggtttac cggtgtcatt ccgctgttat ggccgcgttt gtctcattcc acgcctgaca
15421 ctcagttccg ggtaggcagt tcgctccaag ctggactgta tgcacgaacc ccccgttcag
15481 tccgaccgct gcgccttatc cggtaactat cgtcttgagt ccaacccgga aagacatgca
15541 aaagcaccac tggcagcagc cactggtaat tgatttagag gagttagtct tgaagtcatg
15601 cgccggttaa ggctaaactg aaaggacaag ttttggtgac tgcgctcctc caagccagtt
15661 acctcggttc aaagagttgg tagctcagag aaccttcgaa aaaccgccct gcaaggcggt
15721 tttttcgttt tcagagcaag agattacgcg cagaccaaaa cgatctcaag aagatcatct
15781 tattaagggg tctgacgctc agtggaacga aaactcacgt taagggattt tggtcatgag
15841 attatcaaaa aggatcttca cctagatcct tttaaattaa aaatgaagtt ttaaatcaat
15901 ctaaagtata tatgagtaaa cttggtctga cagttaccaa tgcttaatca gtgaggcacc
15961 tatctcagcg atctgtctat ttcgttcatc catagttgcc tgactccccg tcgtgtagat

16021 aactacgata cgggagggct taccatctgg ccccagtgct gcaatgatac cgcgagaccc
16081 acgctcaccg gctccagatt tatcagcaat aaaccagcca gccggaaggg ccgagcgcag
16141 aagtggtcct gcaactttat ccgcctccat ccagtctatt aattgttgcc gggaagctag
16201 agtaagtagt tcgccagtta atagtttgcg caacgttgtt gccattgctg caggcatcgt
16261 ggtgtcacgc tcgtcgtttg gtatggcttc attcagctcc ggttcccaac gatcaaggcg
16321 agttacatga tcccccatgt tgtgcaaaaa agcggttagc tccttcggtc ctccgatcgt
16381 tgtcagaagt aagttggccg cagtgttatc actcatggtt atggcagcac tgcataattc
16441 tcttactgtc atgccatccg taagatgctt ttctgtgact ggtgagtact caaccaagtc
16501 attctgagaa tagtgtatgc ggcgaccgag ttgctcttgc ccggcgtcaa cacgggataa
16561 taccgcgcca catagcagaa ctttaaaagt gctcatcatt ggaaaacgtt cttcggggcg
16621 aaaactctca aggatcttac cgctgttgag atccagttcg atgtaaccca ctcgtgcacc
16681 caactgatct tcagcatctt ttactttcac cagcgtttct gggtgagcaa aaacaggaag
16741 gcaaaatgcc gcaaaaaagg gaataagggc gacacggaaa tgttgaatac tcatactctt
16801 cctttttcaa tattattgaa gcatttatca gggttattgt ctcatgagcg gatacatatt
16861 tgaatgtatt tagaaaaata aacaaatagg ggttccgcgc acatttcccc gaaaagtgcc
16921 acctgacgtg tcgacgcggc cgcacattga ttattgacta gttattaata gtaatcaatt
16981 acggggtcat tagttcatag cccatatatg gagttccgcg ttacataact tacggtaaat
17041 ggcccgcctg gctgaccgcc caacgacccc cgcccattga cgtcaataat gacgtatgtt
17101 cccatagtaa cgccaatagg gactttccat tgacgtcaat gggtggagta tttacggtaa
17161 actgcccact tggcagtaca tcaagtgtat catatgccaa gtccgccccc tattgacgtc
17221 aatgacggta aatggcccgc ctggcattat gcccagtaca tgaccttacg ggactttcct
17281 acttggcagt acatctacgt attagtcatc gctattacca tggtgatgcg gttttggcag
17341 tacaccaatg ggcgtggata gcggtttgac tcacggggat ttccaagtct ccaccccatt
17401 gacgtcaatg ggagtttgtt ttggcaccaa aatcaacggg actttccaaa atgtcgtaat
17461 aaccccgccc cgttgacgca aatgggcggt aggcgtgtac ggtgggaggt ctatataagc
17521 agagctcgtt tagtgaaccg
//

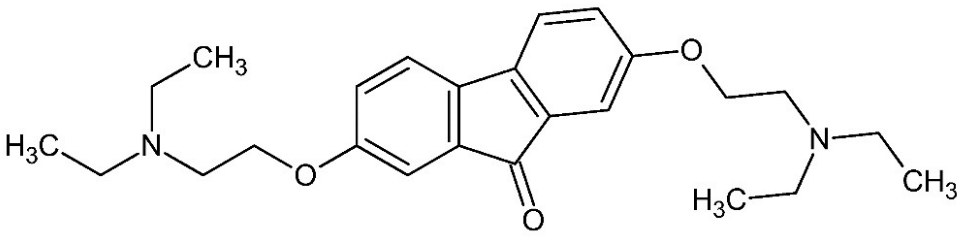

2 HCl

2,7-bis[2-(diethylamino)ethoxy]fluoren-9-one hydrochloride

Tilorone

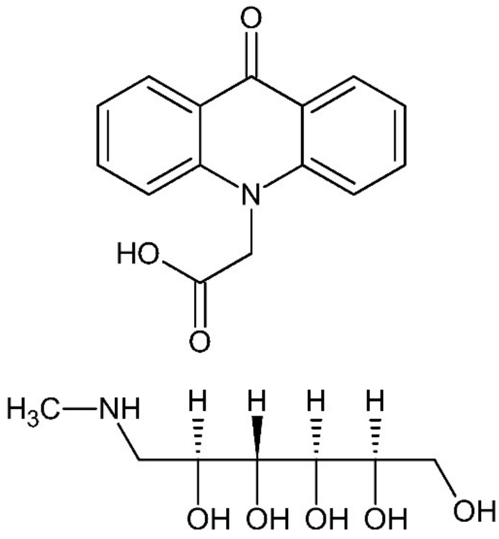

10-carboxymethyl-9-acridanone meglumine salt

Cycloferon

Figure S8. Chemical structures of Tilorone and Cridanimod.

Table S1. Data for calculation of LD50 for the TC-83/TrD virus [1]

Virus dilution	Mice in group	Numbers dead (+) or alive (-)		Cumulative sums		Cumulative percent of dead
		+	-	+	-	
1:10	10	10	0	48	0	100
$1:10^2$	10	10	0	38	0	100
$1:10^3$	10	8	2	28	2	93
$1:10^4$	10	8	2	20	4	83
$1:10^5$	10	8	2	12	6	67
$1:10^6$	10	4	6	4	12	25
$1:10^7$	10	0	10	0	22	0
$1:10^8$	10	0	10	0	32	0
$1:10^9$	10	0	10	0	42	0
$1:10^{10}$	10	0	10	0	52	0

[1] Comments: Adult mice were infected with the TC-83/TrD virus using subcutaneous route and inoculum volume 100 ul. The titer in LD50 units is 2.5×10^6. The same sample titer in PFU is 2.5×10^7 PFU/ml, translating into 1 LD50 = 10 PFU.
